# In vivo subchronic effects of ciguatoxin-related compounds, reevaluation of their toxicity

**DOI:** 10.1007/s00204-022-03315-0

**Published:** 2022-06-03

**Authors:** Sandra Raposo-García, Andrea Boente-Juncal, Mercedes Rodriguez-Vieytes, Mercedes Camiña, Celia Costas, Alejandro Cao, M. Carmen Louzao, Manuel Cifuentes, Carmen Vale, Luis M. Botana

**Affiliations:** 1 Departamento de Farmacología, Farmacia y Tecnología Farmacéutica, Facultad de Veterinaria, Universidad de Santiago de Compostela, Campus Universitario s/n, 27002 Lugo, Spain; 2 Departamento de Fisiología, Facultad de Veterinaria, Universidad de Santiago de Compostela, Campus Universitario s/n, 27002 Lugo, Spain; 3 Departamento de Anatomía, Producción Animal y Ciencias Clínicas Veterinarias, Facultad de Veterinaria, Universidad de Santiago de Compostela, Campus Universitario s/n, 27002 Lugo, Spain

**Keywords:** Maitotoxin 1, Ciguatoxin, Gambierone, Maitotoxin 3 (MTX3) or 44-methylgambierone, Ciguatera fish poisoning, In vivo, Acute toxicity, Subchronic toxicity

## Abstract

Ciguatoxins are marine compounds that share a ladder-shaped polyether structure produced by dinoflagellates of the genus *Gambierdiscus* and *Fukuyoa*, and include maitotoxins (MTX1 and MTX3), ciguatoxins (CTX3C) and analogues (gambierone), components of one of the most frequent human foodborne illness diseases known as ciguatera fish poisoning. This disease was previously found primarily in tropical and subtropical areas but nowadays, the dinoflagellates producers of ciguatoxins had spread to European coasts. One decade ago, the European Food Safety Authority has raised the need to complete the toxicological available data for the ciguatoxin group of compounds. Thus, in this work, the in vivo effects of ciguatoxin-related compounds have been investigated using internationally adopted guidelines for the testing of chemicals. Intraperitoneal acute toxicity was tested for maitotoxin 1 at doses between 200 and 3200 ng/kg and the acute oral toxicity of Pacific Ciguatoxin CTX3C at 330 and 1050 ng/kg and maitotoxin 1 at 800 ng/kg were also evaluated showing not effects on mice survival after a 96 h observation period. Therefore, for the following experiments the oral subchronic doses were between 172 and 1760 ng/kg for gambierone, 10 and 102 ng/kg for Pacific Ciguatoxin CTX3C, 550 and 1760 ng/kg for maitotoxin 3 and 800, 2560 and 5000 ng/kg for maitotoxin 1. The results presented here raise the need to reevaluate the in vivo activity of these agents. Although the intraperitoneal lethal dose of maitotoxin 1 is assumed to be 50 ng/kg, without chemical purity identifications and description of the bioassay procedures, in this work, an intraperitoneal lethal dose of 1107 ng/kg was obtained. Therefore, the data presented here highlight the need to use a common procedure and certified reference material to clearly establish the levels of these environmental contaminants in food.

**Supplementary Information:**

The online version contains supplementary material available at 10.1007/s00204-022-03315-0.

## Introduction

*Gambierdiscus* and *Fukuyoa* species are marine dinoflagellates producers of toxins causative of a widespread human illness known as Ciguatera Poisoning (CP) which includes carnivorous fish that contribute with the higher incidence to Ciguatera Fish Poisoning (CFP) and toxic compounds accumulated by marine invertebrates that also accumulate ciguatoxins but the incidence is minor (Chinain et al. 2021). This syndrome comprises gastrointestinal, neurological, and cardiovascular symptoms (Lehane and Lewis 2000). Blooms of these dinoflagellates have expanded worldwide reaching even European coasts (Canals et al. 2021; de Haro et al. 2021; Katikou 2021; Murray et al. 2021). The presence of *Gambierdiscus* species (Canals et al. 2021; Katikou 2021), the related toxins (Tudó et al. 2020) and CFP intoxications have been repetitively identified in Europe during the last decades (Canals et al. 2021; Katikou 2021), especially in the Canary Islands (Canals et al. 2021; Perez-Arellano et al. 2005), Madeira (Otero et al. 2010) and France (de Haro et al. 2021). CFP is one of the most common forms of seafood poisoning, caused by the consumption of fish contaminated with ciguatoxins (CTXs) produced by microalgae of the genus *Gambierdiscus* (Bagnis et al. 1979) and *Fukuyoa* (Gomez et al. 2015). CTXs enter into the food chain when herbivorous fish consume dinoflagellates and bioaccumulate toxins along the food chain until the compounds reach the apex of the trophic pyramid where humans experience full effects of its bioaccumulation (Goater et al. 2011). Global incidence rates of CFP may arise up to 50,000 people annually, although under-diagnosis and under-reporting occur. Distribution of CFP is boosted paralleling a worldwide increase in harmful algae bloom (HAB) events, international trade expansion and anthropogenic global warming (Botana 2016).

Marine dinoflagellates of the genus *Gambierdiscus* produce several bioactive polyether compounds such as maitotoxins (MTXs), ciguatoxins, gambierol (Lewis 2001; Pearn 2001; Watters 1995; Yasumoto 2001) and the newly identified ladder-shapped polyethers gambierone (Rodriguez et al. 2015) and MTX3 also named 44-methylgambierone (Boente-Juncal et al. 2019a). The implications of all of these compounds in CFP have been suggested by several authors and lead to gastrointestinal, cardiovascular and neurological symptoms in humans that vary among patients and include paresthesia, generalized pruritus, painful dysesthesias, allodynia, ataxia and hyperalgesia (Botana 2016; Murray et al. 2021, 2020; WHO 2020). Traditionally CFP was typically found only in tropical and subtropical areas but during the last decade has expanded to other regions including Europe (Canals et al. 2021; de Haro et al. 2003; EFSA 2010; Katikou 2021), a fact that raises the need to establish regulations for the levels of these food contaminants.

Besides ciguatoxins, which can cause long-term neurological sequela in humans as a consequence of their permanent activation of voltage-gated sodium channels (Benoit et al. 1986; Hidalgo et al. 2002; Martin et al. 2015a, b; Molgo et al. 1990), the structure of an additional ciguatoxin-congener, named MTX3, has been recently elucidated (Boente-Juncal et al. 2019a). Initial studies on the in vitro biological activity of MTX3described an effect similar to that of the synthetic ciguatoxin CTX3C although of much lower potency (Boente-Juncal et al. 2019a) and with a LD_50_ in vivo between 20 and 38 mg/kg when administered by the intraperitoneal (i.p.) route (Murray et al. 2020) and of 3.8 mg/kg when algal extracts containing MTX3 were administered intraperitoneally after extraction from 4.776 × 10^6^ cells of *G. belizeanus* CCMP401 (Kohli et al. 2015). Gambierone is another compound thought to be associated with CFP, since it is routinely found in dinoflagellates producers of ciguatoxins (Rodriguez et al. 2015) that has a ciguatoxin-like activity but low i.p. potency, with a LD_50_ of 2.4 mg/kg after i.p administration of the compound purified from *G. cheloniae* CAWD232 monoclonal cultures (Murray et al. 2021). Regarding MTX1, this compound was historically assumed to be one of the more toxic natural compounds with an i.p. LD_50_ in mice of 50 ng/kg, described initially after the structure elucidation of this toxin (Murata et al. 1993) but i.p. administration to mice of *Gambierdicus australes* samples, containing a combination of MTX1 and 44-methylgambierone, led to a LD_50_ between 200 and 1060 ng/kg (Munday et al. 2017), while in the same report administration of the algal extracts by gavage gave a LD_50_ between 20,000 and 84,200 ng/kg. The discrepancies among these values prompted us to further evaluate the toxicity of the ciguatera-related compounds following internationally adopted guidelines for the testing of chemicals (OECD 2008a; b).

With the aim of ultimately set the oral subchronic toxicity of the ciguatoxin-related compounds involved in CFP, first, the acute i.p. toxicity of MTX1 as well as an approach to the acute oral toxicity of MTX1 and CTX3C were carried out to establish the posterior doses to be administered by gavage during a 28-day period (OECD 2008a) and thus determine the oral toxicity of the compounds evaluated. The data presented evaluated the daily subchronic exposure of mice to different toxins responsible of CFP following internationally adopted guidelines and blood and urine biochemistry parameters. Moreover, the acute i.p. toxicity of MTX1 was about 20 times lower than that previously described, even using a 96-h observation period. A fact that is consistent with the observation of no lethality of the toxin even at daily oral doses of 5000 ng/kg.

## Materials and methods

### Toxins and drugs used

MTX1 and CTX3C both purity higher than 99%, were obtained from Wako (Fujifilm Wako Pure Chemical Corporation, Japan) and dissolved at a concentration of 10 μM in dimethyl sulfoxide (DMSO). Immediately before administration, MTX1 was diluted in physiological buffer solution, to a concentration of 5 µM to obtain the series of tested doses. Working solutions of CTX3C from a stock solution of 1 µM were also prepared. Gambierone was purchased from CIFGA S.A. (Lugo, Spain), the stock solution was 157.4 µM and was diluted to 10 µM in DMSO. MTX3 was also supplied from CIFGA with a stock solution of 164.4 µM and working solutions were prepared in DMSO at a final concentration of 10 µM. Control animals received the same amount of physiological saline solution that contained the corresponding amount of toxin solvent, always lower than 1% (v/v) either for the oral route (0.9% at the highest MTX1 dose) or for the i.p. administration (0.84%).

### In vivo experimental procedure

In vivo studies were performed with Swiss male and female mice weighting of body weight (bw) ranging from 22 to 26 g and 4 weeks old at the beginning of the experiments. All in vivo procedures were performed in compliance to the European legislation (EU directive 2010/63/EU) and according to the Spanish legislation (Real Decreto 53/2013). The principles were approved by the Institutional Animal Care Committee of the Universidad de Santiago de Compostela under the procedure number 011/14, authorized on August 14th 2014 (MR110250) and under procedure number 06/19/LU-002, authorized on March 1st, 2019 (A12X00509).

Before administration, the stocks of CTX3C, gambierone, MTX1 and MTX3 were diluted in 0.9% saline solution in a final volume of 200 µl. For acute studies, animals received either a single oral dose of toxin by gavage or, in the case of MTX1, a single i.p. injection. The same procedure was followed for control animals. The i.p. doses for MTX1 were 200, 400, 800, 1000, 1200, 1600 and 3200 ng/kg body weight while the oral acute dose was 800 ng/kg bw for MTX1 and 330 and 1050 ng/kg bw for CTX3C. No acute studies were performed for MTX3 and gambierone since previous works highlighted their much lower toxicity (Murray et al. 2020, 2021). After toxin administration, mice were observed continuously for the first 2 h and periodically afterwards until sacrifice of surviving animals at the end of the treatments. During the procedures, moribund animals or mice obviously in pain or presenting signs of severe agony were humanely killed and considered in the interpretation of the test results as animals that died during the experiment.

For the repeated oral dosing, toxins were administered in three separate treatments and animal groups, each lasting 28 days, starting at the lower dose and initiating the next dosage period after finishing the first month treatment. To create toxin groups and control groups, animals were randomly selected, marked to allow identification, and kept in their cages for 5 days to acclimatize before the beginning of the experiments, as required by the current protocols (OECD 2008b). In the oral treatments CTX3C at doses of 10, 32, 102 ng/kg bw was administrated by gavage every 24 h. Gambierone administration to mice included doses of 172, 550 and 1760 ng/kg bw while MTX1 was fed to mice at doses of 800, 2560 and 5000 ng/kg bw. Finally, MTX3 was daily administrated to mice at doses of 550 and 1760 ng/kg bw. During the 28-day treatment period, animals were housed in rooms with controlled temperature, humidity, and a restricted photoperiod at the animal facilities of the School of Veterinary Medicine of the University of Santiago de Compostela (Code: AE-LU-002). The toxins doses and the routes of administration used in each case are summarized in Table 1.

**Table 1:** Toxin doses and administration routes evaluated in this work

Toxin doses (ng/kg)	MTX1	CTX3X	Gambierone	MTX3
Acute i.p	200, 400, 800, 1000, 1200, 1600, 3200			
Acute oral	800	330, 1050		
28-day oral subchronic	800, 2560, 5000	10, 32,102	172, 550, 1760	550, 1760

For the repeated oral treatment, control group and toxin-treated mice groups were weighted the first day of the treatment (day 0) and weekly thereafter. Food consumption was also recorded at days 7, 14, 21 and 28 from the beginning of the treatments. Euthanasia was performed on day 28, and 24 h before sacrifice animals were individually placed in metabolic cages to monitor and obtain samples of urine and feces production during this period. For all the treatments animals were euthanized in a CO_2_ chamber and directly, after death confirmation by cervical dislocation, blood and tissue samples were collected. Mice were subjected to a detailed necropsy including observation of thoracic organs (heart and lung), abdominal organs (liver, kidneys, spleen, stomach, duodenum, rectum) and brain.

### Evaluation of enzyme and electrolyte levels in plasma of control and toxin-treated animals

Blood was obtained directly from the ventricle of euthanized mice as previously described (Boente-Juncal et al. 2020) and conserved in microtubes containing lithium heparin (2.5 UI/ml) until the end of the necropsy and tissue collection (30 min). Afterwards, each blood sample was mixed for 30 s and centrifugated for 90 s at 15,800 rpm/12000×*g* (IDEXX Stat Spin, IDEXX Europe B.V., Hoofddorp, The Netherlands). The computer controlled biochemical analyzer IDEXX Catalyst Dx was used for the analysis of plasma parameters. In most of the samples, 300 μL of plasma were used to evaluate electrolyte levels (Cl^−^, Na^+^ and K^+^), alanine transaminase (ALT), aspartate transaminase (AST), lactate dehydrogenase (LDH), and creatine kinase (CK). Initially, all the markers were evaluated in undiluted samples, but when the plasma level of the marker was above the reference range of the analyzer, an automatic dilution with physiological saline was performed. Samples with altered visual properties that could alter biochemical parameters (high hemolysis, icterus or lipidemia) were not further processed.

### Urine analysis

Parameters as urine color, turbidity, protein, glucose, ketones, blood hemoglobin, bilirubin, and urobilinogen were analyzed using an IDEXX VetLab UA reflectance photometer, which reads and evaluates IDEXX UA Strips. Urine specific gravity was measured with a refractometer. Because for some mice a small amount of blood or urine were obtained, it was not possible to measure all the parameters for each animal.

### Preparation of samples for light microscopy

After sacrifice, necropsy and macroscopic examination of the different organs, samples were prepared for light microscopy. Tissue samples were fixed by immersion in buffered Bouin’s solution (75% picric acid, 20% formaldehyde, 5% acetic acid) for 24 h at 4 °C, transferred to 70% alcohol and embedded in paraffin wax according to standard laboratory procedures. Tissue samples were cut into sections of 3 µm thickness and mounted into slides. Sections were stained with haematoxylin–eosin (H&E) for structural assessment of the tissues and examined under a light microscope. Digitalized images were obtained with an Olympus microscope digital camera DP74 coupled to an Olympus AX70 microscope.

### Statistical analysis

Data analysis was performed using GraphPad Prism 5. One-way ANOVA followed by Bonferoni *t* test or Student’s *t* test were used to assess significant differences between control and SPX-treated neurons. All values are expressed as mean ± SEM of at least three independent experiments.

## Results

In this in vivo study, an acute and repeated oral dosing evaluation of the effect of several compounds involved in ciguatera fish poisoning was performed. First, the acute toxicity of the most representative analogues (MTX1 and CTX3C) was evaluated using a single approach to elucidate the optimum doses that could give consistent results for the 28-day oral approach. Although MTX1 was so far considered one of the most toxic compounds known to date, with a reported initial i.p. LD_50_ in mice of 50 ng/kg (Murata et al. 1993), a much higher LD_50_ (approximately 0.2 mg/kg) for algal extracts containing a mixture of MTX1 and MTX3 was reported after a 96-h observation period (Munday et al. 2017). Therefore, this study was initiated following the same approach, using first MTX1 due to its assumed high toxicity by the i.p. route (Murata et al. 1993).

### Acute i.p. toxicity of MTX1

**Fig. 1:**
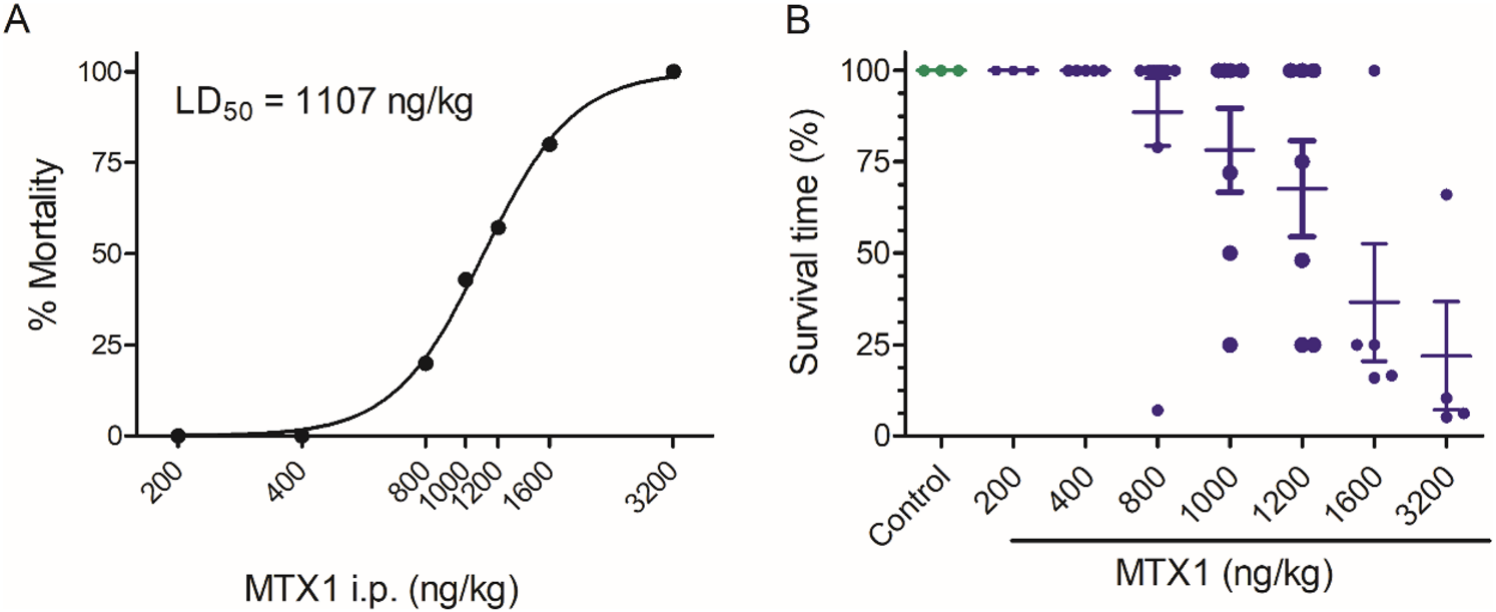
Intraperitoneal toxicity of MTX1 after a 96-h observation period. **A** Lethality of MTX1 in Swiss female mice after injection of different toxin doses. **B** Scatter plot graph presenting the percentage of survival time for each mouse. The survival results are expressed as means ± SEM of the data obtained from three to ten animals after an observation period of 96 h

To optimize the dose of MTX1 needed to establish the optimum quantities of toxin to evaluate its oral toxicity, an initial approach was performed evaluating the effect of the i.p. injection of MTX1. The effects of i.p. administration doses of MTX1, ranging from 200 to 3200 ng/kg, after a 96-h observation period and the corresponding survival times for each animal are summarized in Table 2. Non-linear fit of the mortality of animals after i.p. administration of increasing MTX1 (Fig. 1A) allowed to calculate an estimated i.p. LD_50_ for MTX1 of 1107 ng/kg [95% confidence interval (CI) from 1075 to 1140 ng/kg, *R*^2^ = 0.9981]. A scatter plot showing the relative survival times for each mouse at the different MTX1 doses are shown in Fig. 1B.

**Table 2:** Mortality induced by single i.p. administration of MTX1 to mice and survival times corresponding with each treatment

Dose (ng/kg)	Mice number	Dead	Survival time (h)	Mortality %
Control	4	0	96	0
MTX1				
200	3	0	96	0
400	5	0	96	0
800	10	2	3.5, 34, 96, 96, 96, 96, 96, 96, 96, 96	20
1000	7	3	24, 48, 72, 96, 96, 96,96	42.8
1200	7	4	24, 24, 48, 72, 96,96,96	57.1
1600	5	4	4, 16, 24, 24, 96	80
3200	4	4	2.5, 5, 6, 32	100

The symptoms shown by the animals after acute i.p. administration of the different MTX1 doses are summarized in Table 3. Most of the animals showed lethargy and piloerection even with low doses of i.p. MTX1 (200 and 400 ng/kg). Stretching and Straub´s tail reaction (dorsiflexion of the tail orientated vertical or curling over the mouse produced by contraction of the sacrococcygenus muscle, (Supplementary Figs. 1A and B) were observed in mice after administration of the toxin at 400 ng/kg and higher, an effect whose intensity and duration was dependent on the toxin dose. At higher doses of MTX1, ataxia and kyphosis were also observed. Furthermore, MTX1 at 800 ng/kg and higher caused dyspnea that led to a profound abdominal breathing that was evident in most animals. The dyspnea was justified after opening the thoracic cavity of the moribund animals, that revealed accumulation of blood and internal bleeding. Also, an intense cyanosis of the tail in five of the animals was observed after the first 48 h of i.p. administration of the toxin. Dyspnea was observed in all the animals treated with the highest MTX1 dose.

**Table 3:** Main symptoms elicited in mice after acute i.p. administration of MTX1 and number of animals with symptoms at each MTX1 dose

I.p. MTX dose (ng/kg)	200	400	800	1000	1200	1600	3200
Lethargy	1/3	5/5	10/10	7/7	7/7	5/5	4/4
Piloerection	1/3	2/5	7/10	7/7	7/7	5/5	4/4
Stretching	2/3	5/5	5/10	7/7	7/7	5/5	4/4
Straub tail reaction	0/3	2/5	5/10	5/7	7/7	5/5	4/4
Ataxia	0/3	2/5	7/10	5/7	7/7	5/5	4/4
Kyphosis	0/3	0/5	2/10	5/7	5/7	5/5	0/4
Dyspnea	0/3	0/5	4/10	5/7	5/7	3/5	4/4
Cyanosis	0/3	0/5	1/10	2/7	2/7	2/5	0/4
Abdominal swelling	0/3	0/5	5/10	7/7	7/7	5/5	4/4

**Fig. 2:**
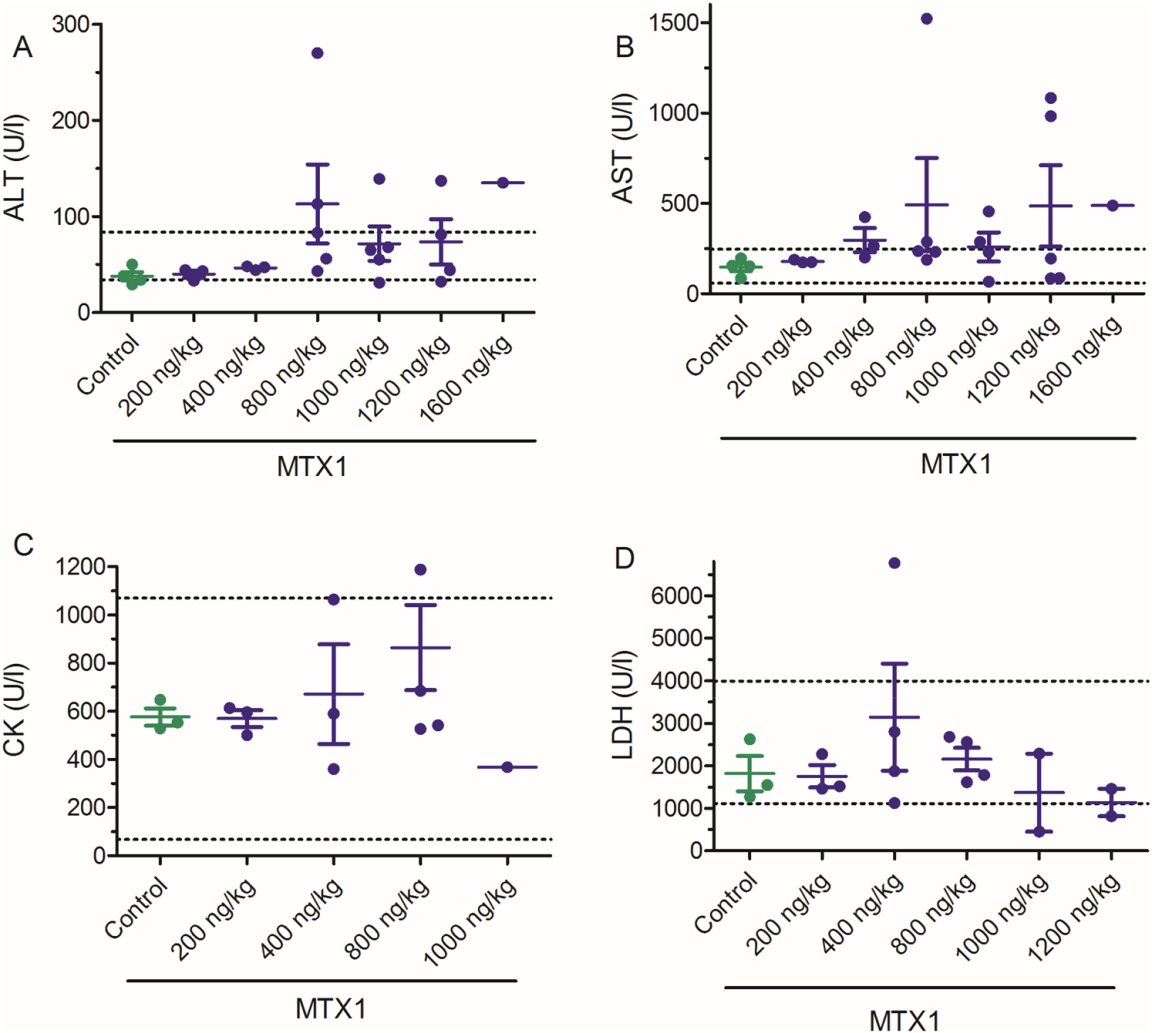
Scatter plot graphs showing the effects of different i.p. doses of MTX1 (200, 400, 800, 1000, 1200 and 1600 ng/kg), on blood levels of ALT, AST, CK and LDH. Data are expressed as mean ± SEM from at least two animals. The physiological ranges for the blood level of each enzyme are indicated by the dotted lines

As shown in Fig. 2, single i.p. administration of MTX1 increased several blood enzyme levels in respect to control animals although most of the parameters were still within the normal physiological range. Figure 2 summarizes the biochemical levels of the different parameters in control and MTX1-treated mice. Although ALT levels were increased in some of the animals treated with MTX1, the differences between control and toxin-treated animals did not reach statistically significant differences at any of the toxin doses employed (Fig. 2A). Blood enzyme levels were not analyzed in mice treated with MTX1 at doses of 1600 and 3200 ng/kg due to the sudden death of the animals after the first continuous 2 h of the observation period. Similarly for the blood AST levels shown in Fig. 2B, i.p. administration of MTX1 (Fig. 2B) In addition, no statistically significant changes were observed neither for LDH nor CK parameters (Fig. 2C and D, respectively).

**Fig. 3:**
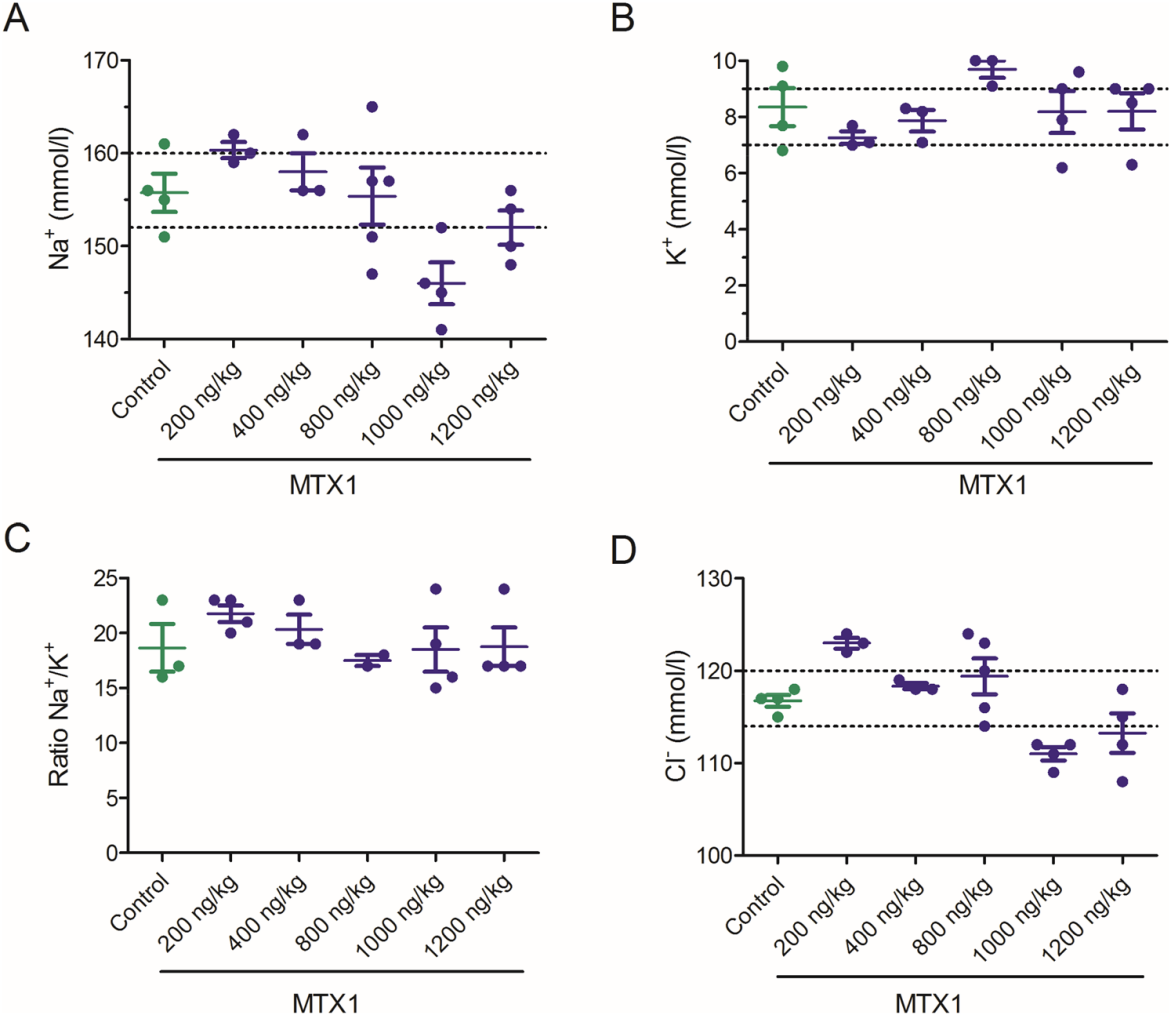
Scatter plot graphs showing the effects of different i.p. doses of MTX1 on blood electrolyte levels. Data are expressed as mean ± SEM. The respective minimum and maximum reference values of blood electrolyte levels are indicated by the dotted lines

Simultaneously, the effect of the i.p. administration of MTX1 on blood electrolyte levels was evaluated as shown in Fig. 3, but no statistically significant differences on blood ion levels were found between control animals and animals treated intraperitoneally with different doses of MTX1.

After euthanasia, animals were subjected to macroscopic observation on necropsy. As shown in Supplementary Fig. 2, macroscopic alterations were present in animals treated at doses of 1000 ng/kg MTX1 and higher. All animals treated with these doses of MTX1 had the stomach and small intestines empty of solid contents only having feces in the final sections of the large intestine. It is noteworthy to remark that despite abdominal swelling, all treated animals had a remarkable weigh loss (between 1 and 3.6 g). Thus, abdominal distension was observed in the gastrointestinal tract especially in the stomach, small intestine and caecum due to gas accumulation in MTX-treated animals. Additionally, two animals treated with MTX1 at doses of 1200 ng/kg presented several haemorrhagic zones in the stomach observed macroscopically as shown in Supplementary Fig. 3.

**Fig. 4:**
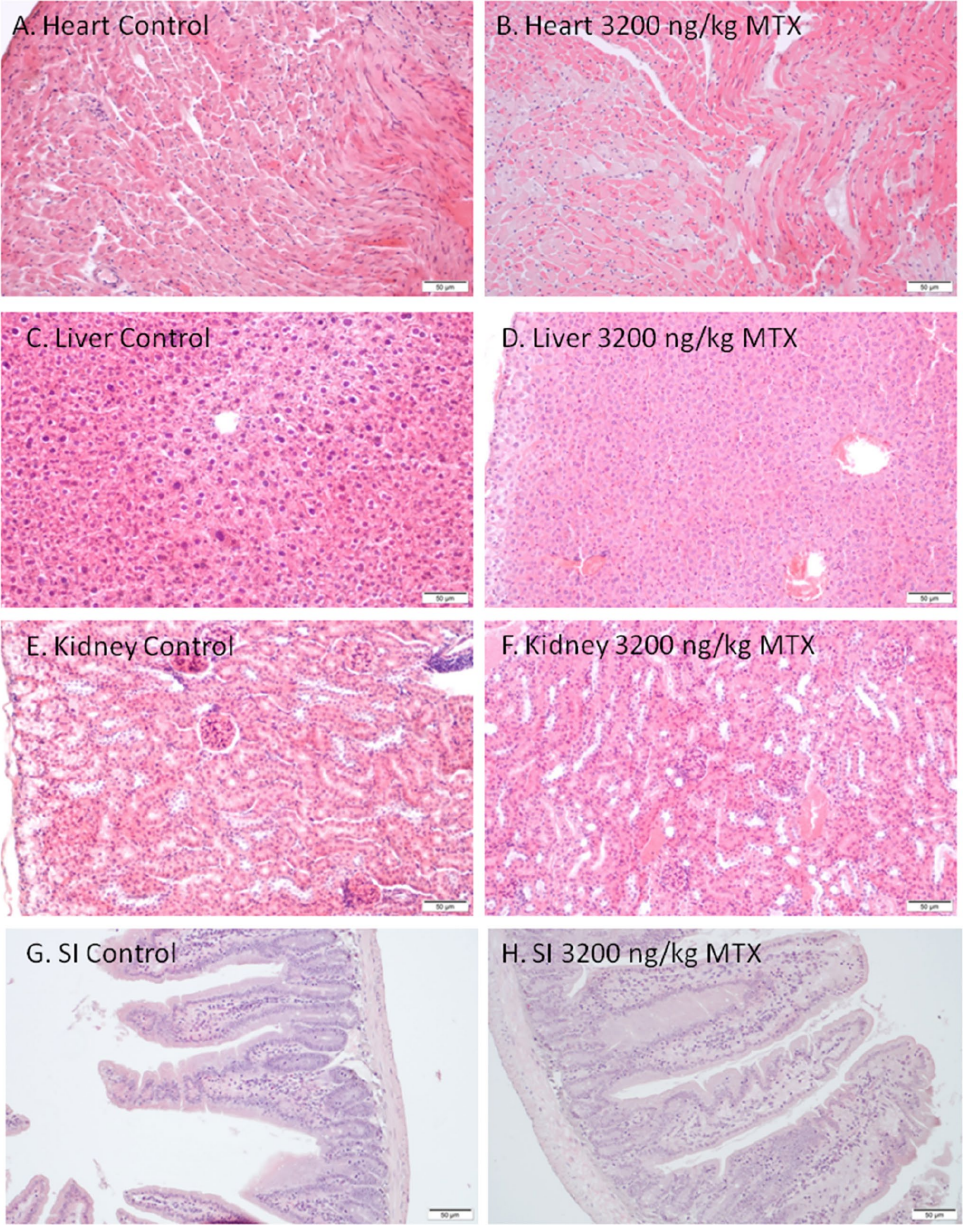
Light microscopy images of heart, liver, kidney and small intestine of a control mouse (**A**, **C**, **E**, **G**) and an animal dosed intraperitoneally with 3200 ng/kg of MTX1 that survived 32 h (**B**, **D**, **F**, **H**)

Images of different tissues (20×) stained with H&E for structural assessment under light microscopy are represented in Fig. 4. As shown in the corresponding images, histological examination of the heart of control and MTX mice dosed with 3200 ng/kg of MTX1 (Figs. 4A and B, respectively) that survived for 32 h did not show any evidence of structural alterations. In both cases, normal cylindrical-shaped cardiac myocytes with elongated nuclei, were observed. Similarly, liver sections (Fig. 4C and D) of control and MTX1-treated mice showed an eosinophilic cytoplasm with large spherical and central nuclei, which corresponds to a normal morphology. The kidney sections from control animals and from MTX1-treated mice (Fig. 4E and F) did not reveal any apparent histological alterations by light microscopy. Small intestine of control and MTX1-treated mice (Fig. 4G and H) did not show any apparent changes in the arrangement of plicae around the lumen or in the surrounding musculature. Large intestine samples were also stained and observed but had unaltered basic structure formed by the mucosa and the submucosa layer, the smooth muscle layers, and the serosa layer both in control and MTX1-treated mice (data not shown). Since this mouse only survived 32 h, samples of the same tissues of mice dosed intraperitoneally with 800 ng/kg of MTX1 that survived the 96-h observation period are also shown in Supplementary Fig. 4.

### Acute oral evaluation of the toxicity of CTX3C and MTX1

After confirming that a single i.p. dose of 800 ng/kg of MTX1 did not elicit drastic changes in blood parameters, initially the same toxin dose was evaluated by the oral route administrating the toxin by gavage and comparing the effect of MTX1 with the effects elicited by CTX3C. Since the cellular effects of MTX1 are much more pronounced than those of CTX3C (Boente-Juncal et al. 2019a), to establish the toxin doses needed for the subchronic toxicity approach, a single dose of 800 ng/kg of MTX1 was orally administrated to three animals. Additionally, CTX3C at doses of 330 and 1050 ng/kg was evaluated in the acute toxicity study to establish a reference range prior to start the repeated oral exposure of mice to the toxins since the initial acute LD_50_ dose proposed for CTX3C was approximately 1.5 µg/kg (Hirama et al. 2001). As indicated in Table 4, neither MTX1 nor CTX3C caused death of the animals after the 96-h observation period.

**Table 4:** No mortality was induced by single gavage administration of CTX3C or MTX1 to Swiss female mice during the 96-h observation period

Dose (ng/kg)	Mice number	Dead	Survival time (h)	Mortality %
Control	3	0	96	0
CTX3C				
330	3	0	96	0
1050	3	0	96	0
MTX1				
800	3	0	96	0

**Fig. 5:**
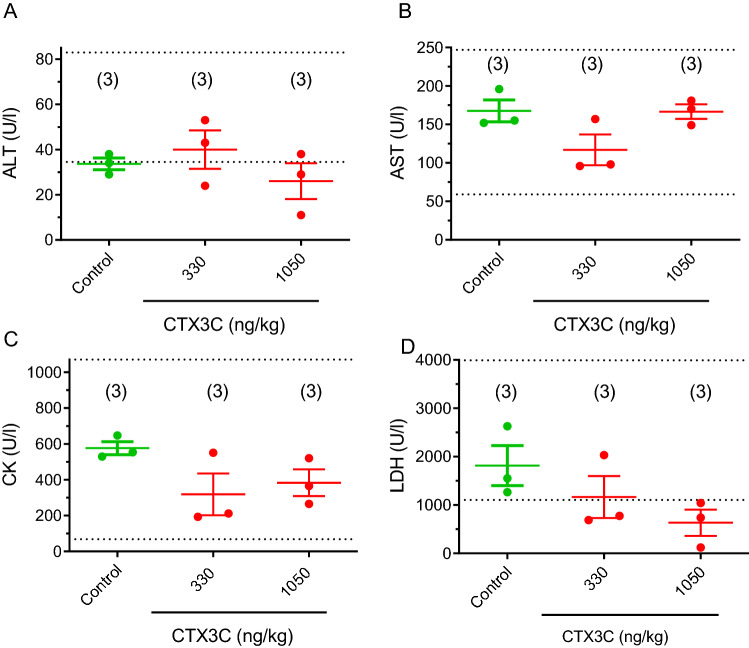
Scatter plot graphs showing the effects of 330 and 1050 ng/kg CTX3C administered by gavage on the blood levels of ALT (**A**), AST (**B**), CK (**C**), and LDH (**D**) after an observation period of 96 h. Data are expressed as means ± SEM from three determinations. The respective minimum and maximum reference values of blood enzyme levels are indicated by the dotted lines

**Fig. 6:**
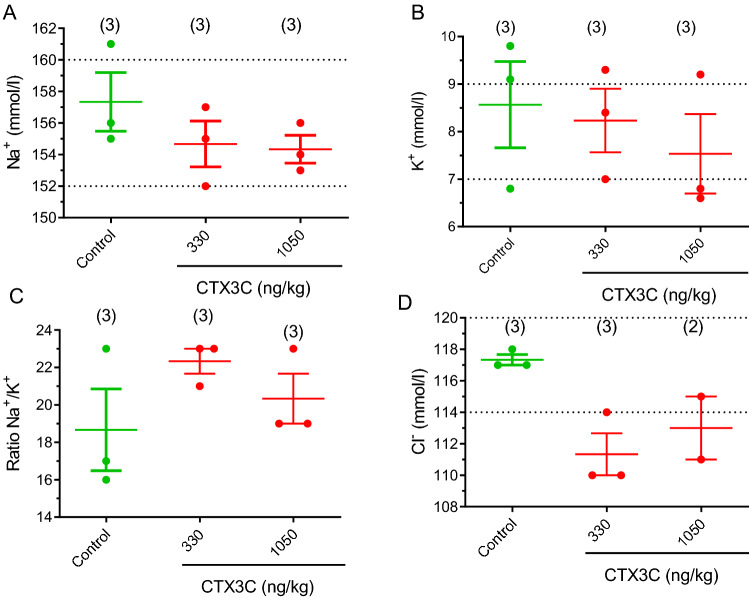
Scatter plot representation of the blood levels of sodium (**A**), potassium (**B**), ratio sodium/potassium (**C**) and chloride (**D**) in control Swiss female mice and in mice dosed by gavage with CTX3C at 330 and 1050 ng/kg bw after an observation period of 96 h. Data are expressed as means ± SEM The dotted line indicates the maximum and minimum physiological reference value for each parameter. Values are expressed as mean ± SEM from three determinations except for determination of Cl^−^ in animals treated with 1050 ng/kg of CTX3C since the size of the sample was not enough in one of the treated animals

No significant effects in blood enzyme levels of ALT, AST, CK and LDH nor in blood electrolyte levels were observed in mice treated with a single oral dose of CTX3C, as shown in Figs. 5 and 6, respectively. Although apparently CTX3C caused a dose-dependent decrease in LDH values the data did not reach statistically significance (*p* = 0.0752; *df* = 4; *t* = 2.389) between control animals and those treated with 1050 ng/kg CTX3C.

In the same animals, the effect of oral acute exposure of mice to CTX3C at concentrations of 330 ng/kg and 1050 ng/kg on food intake, feces and urine production during a 24-h period before euthanasia was measured and is summarized in Table 5 and supplementary Fig. 5. In the case of CTX3C oral administration of the toxin at the dose of 1050 ng/kg decreased urine production in 24 h by about 85% suggesting that either activation or inhibition of voltage-gated sodium channels can alter renal function as previously described for the sodium channel blocker tetrodotoxin (Boente-Juncal et al. 2019b).

**Table 5:** Results obtained after a single oral administration of CTX3C at doses of 330 ng/kg and 1050 ng/kg regarding food consumption, feces, and urine production for 24 h

Group/analyzed parameters (24 h in metabolic cages *n* = 3)	Control	CTX3C 330 ng/kg	CTX3C 1050 ng/kg
Food consumption (g)	3.78 ± 0.42	4.7 ± 0.6	3.7 ± 0.4
Feces (g)	1.57 ± 0.12	1.2 ± 0.3	1.3 ± 0.2
Urine (ml)	4.1 ± 1.44	1.5 ± 0.2*	0.6 ± 0.1**

### Repeated dose effects in mice of the oral administration of toxins involved in ciguatera

Based on the previous results for the oral acute toxicity of MTX1 and CTX3C in mice, the effect of 28-day repeated oral doses of these compounds was explored during a 28-day exposure period. First, since previous studies have shown that intermittent administration of P-CTX1 at 100 ng/kg caused CFP symptoms after 15 days, repeated dosing (Terao et al. 1992), an initial dose of 10 ng/kg CTX3C was chosen. Further doses were increased by the 3.2 rate as indicated in the OECD guidelines for the testing of chemicals (OECD 2008a; b). Therefore, at the end of the study, three independent groups of mice were feed daily by gavage as summarized in Table 1. The effects of the 28-days oral administration of the different toxin doses in mice survival time and mortality rate are summarized in Table 6. As shown in this table MTX1 elicited a dose dependent death, but no toxicity was observed for CTX3C, gambierone or MTX3 at the doses employed. The lowest oral dose of MTX1 was 800 ng/kg since acute oral administration of this toxin dose only elicited piloerection and some degree of immobility during the first 24-h observation period, symptoms that reversed completely after 48 h of feeding the mice by gavage with a single MTX1 dose. During the 28-day treatment period, no changes in body weight in any of the toxin-treated animal groups, were observed as summarized in Supplementary Table 1.

**Table 6:** Mice mortality and survival times observed after daily dosing of Swiss female mice with CTX3C, gambierone, MTX3, or MTX1 during a 28-day period

Dose (ng/kg)	Mice number	Death	Survival time (days)	Mortality %
Control	7	0	28	0
CTX3C				
10	3	0	28	0
32	3	0	28	0
102	3	0	28	0
Gambierone				
172	3	0	28	0
550	3	0	28	0
1760	3	0	28	0
MTX3				
550	2	0	28	0
1760	3	0	28	0
MTX1				
800	3	0	28	0
2560	3	2	5,17,28	66
5000	5	5	1,6,10,14,15	100

**Fig. 7:**
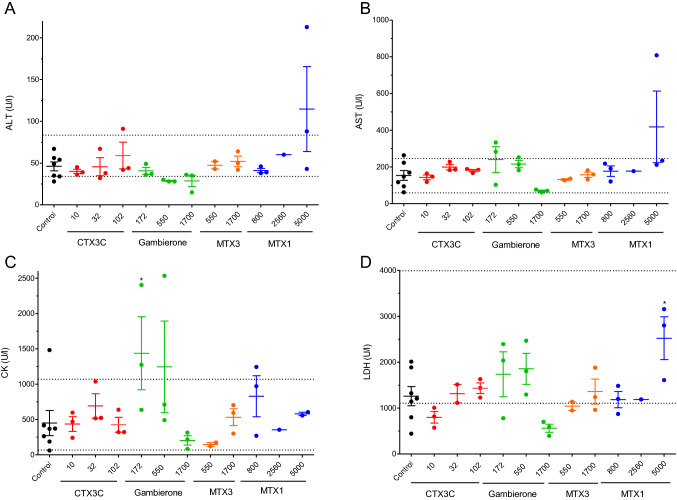
Scatter plot graphs representing blood levels of alanine transaminase (ALT) (**A**), aspartate transaminase (AST) (**B**), creatin kinase (CK) (**C**), and lactate dehydrogenase (LDH) (**D**) in control Swiss female mice and in animals dosed daily by gavage during a 28-day period with CTX3C, gambierone, MTX3 or MTX1 at different doses. *p < 0.05 versus control mice. The respective minimum and maximum reference blood values for each parameter are marked by the dotted lines

As well as in the acute i.p. studies, the enzyme and electrolyte disturbances in the plasma of control and animals treated with toxins for 28 days were analysed and summarized in Fig. 7. None of the toxins caused significative alterations in the hepatic parameters of AST, and ALT (Fig. 7A and B, respectively). Similarly, CK remained within physiological values in all animals treated with toxins except in one of the animals treated with 172 ng/kg of gambierone (Fig. 7C). Moreover, none of the compounds altered the plasma levels of LDH at any of the doses evaluated, and only statistically significant increases in LDH levels were found in mice treated with 5000 ng/kg of MTX1 (Fig. 7D), but the blood LDH levels in MTX1-treated animals were within the physiological range (1.105–3.993 U/L) in all samples.

**Fig. 8:**
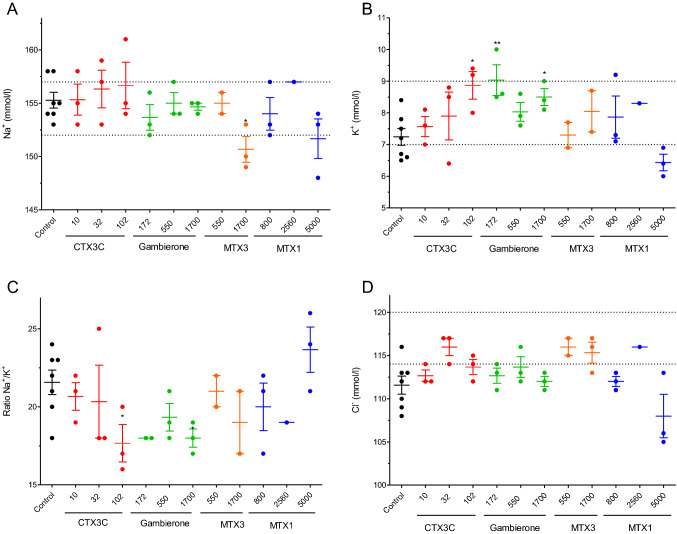
Scatter plot representation of the blood levels of sodium (Na^+^) (**A**), potassium (K^+^) (**B**), ratio sodium/potassium (Na^+^/K^+^) (**C**) and chloride (Cl^−^) (**D**) in control Swiss female mice and in mice dosed daily by gavage, over a 28-day period, with CTX3C, gambierone, MTX3, MTX1. *p < 0.05; **p < 0.01 versus control mice. The respective minimum and maximum reference values of blood electrolyte levels are indicated by the dotted lines

The effects of the repeated oral administration of different doses of CTX3C, gambierone, MTX3 and MTX1 on blood electrolyte levels were also evaluated as shown in Fig. 8. Although none of the toxins affected blood sodium levels (Fig. 8A), in mice fed orally with doses of 10, 32 and 100 ng/kg of CTX3C statistically significant increases in blood potassium levels were found at doses of 100 ng/kg (*df* = 8; *t* = 3.277; *p* = 0.011). As shown in Fig. 8B, the mean K^+^ blood values in control animals were 7.24 ± 0.266 mmol/L (*n* = 7) while in mice fed orally with CTX3C at 100 ng/kg, potassium levels were 8.87 ± 0.44 mmol/L (n = 3). Hence, due to the increase in blood potassium concentration, the ratio Na^+^/K^+^ decreased (Fig. 8C). Regarding the oral treatment with 172, 550 and 1700 ng/kg of gambierone, also statistically significant disturbances in potassium levels as well as alterations in the Na^+^/K^+^ ratio were observed (Fig. 8). Thus, mean K^+^ blood values after the 28 days oral dosing of mice with gambierone at the lowest concentrations evaluated (172 ng/kg) were 9.03 ± 0.48 mmol/L (*n* = 3; *df* = 8; *t* = 3.505; *p* = 0.008) and 8.05 ± 0.65 mmol/L (*n* = 3; *df* = 8; *t* = 2.796; *p* = 0.0234) at the dose of 1700 ng/kg. No alterations in blood electrolyte levels were found in mice treated with MTX3 or with MTX1.

**Fig. 9:**
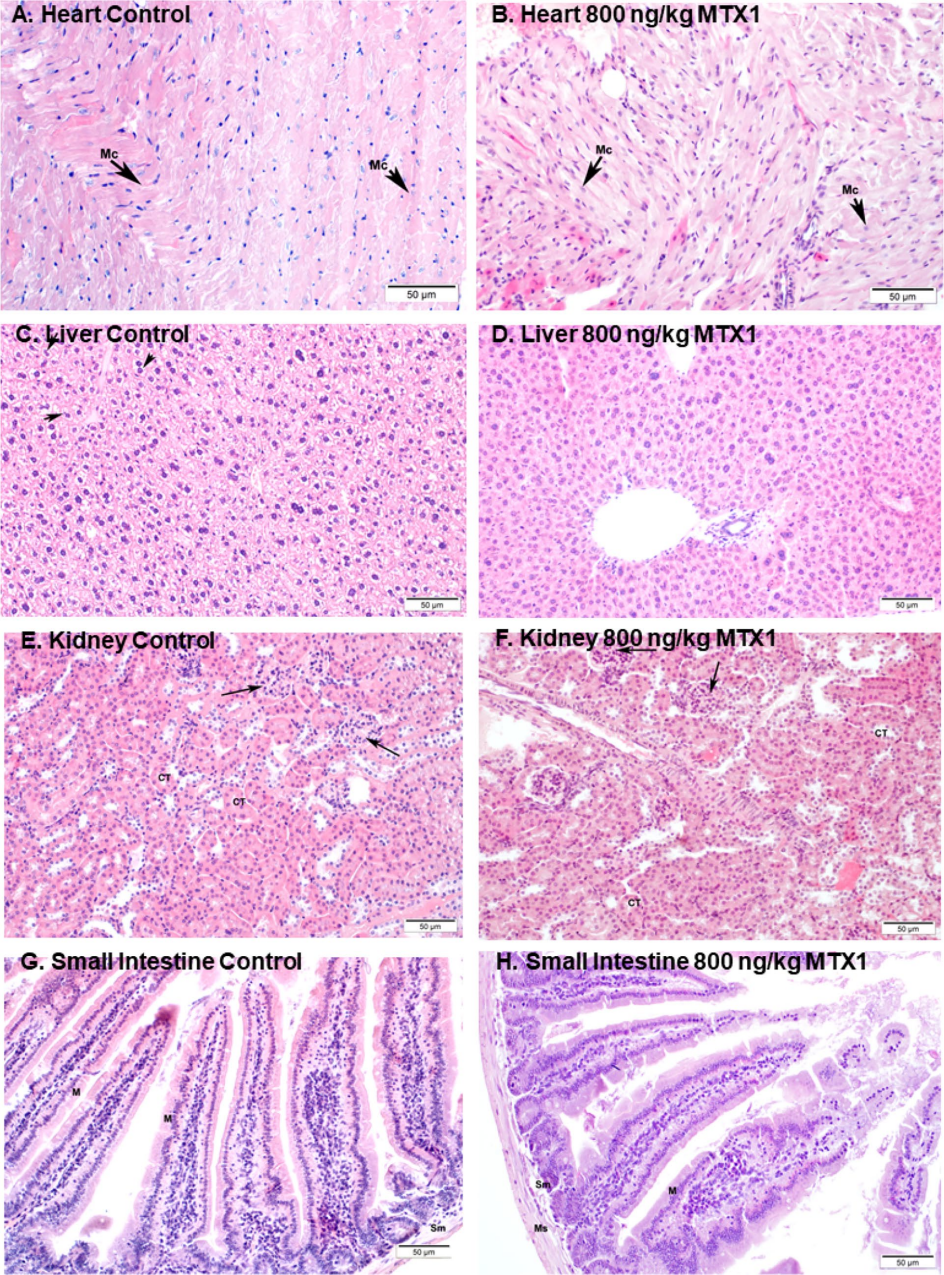
Representative haematoxylin–eosin stained sections of control mice (**A**, **C**, **E** and **G**) and animals treated daily for 28 days with MTX1 at the dose of 800 ng/kg (**B**, **D**, **F** and **H**). Sections of the myocardium ventricle of control (**A**) and treated mouse (**B**) showing normal cardiomyocites (Mc). Sections of the liver parenchyma from a control mouse (**C**), and from a mouse treated with 800 ng/kg MTX1 (**D**). Compared to hepatocytes from MTX-treated animals more cytoplasmic vacuoles (non-stained spaces, indicated by arrows) were present in the hepatocytes of control mice. Kidney sections of a control mouse (**E**) and a mouse treated with 800 ng/kg MTX (**F**). Renal corpuscles (arrows) and convoluted tubules (CT) are shown. Microscope micrographs of the small intestine, from control mice (**G**) and mice treated with MTX at 800 ng/kg (**H**). *M* mucosa layer, *Sm* submucosa layer, *Ms* muscular layer. Microscope magnifications of 20×

As for the acute i.p. treatment, after euthanasia, samples of heart, liver, kidney, and small intestine of control and 800 ng/kg MTX1-treated mice were studied. Representative light microscopy images of H&E staining in heart sections of control and MTX1-treated mice did not show any evidence of structural alterations in the heart (Fig. 9A and B). In both cases, normal cardiac myocytes, which are cylindrical in shape and have an elongated nucleus, were observed. Stained liver samples are shown in Fig. 9C and D. In contrast to liver sections from treated mice, in which hepatocytes showed an eosinophilic cytoplasm and a round nucleus (Fig. 9D), hepatocytes in the livers of control mice showed a more vacuolated (non-stained) cytoplasm (Fig. 9C) that could represent cellular swelling, lipid accumulation, or glycogen stores. These differences may be due to the feeding status of the animal at the time of death. No evident histological alterations in the kidney sections from mice treated with 800 ng/kg MTX1 were observed (Fig. 9E and F). Finally, small intestine showed unaltered mucosa in both control and treated animals and no apparent histological alterations (Fig. 9G and H). Both intestines showed a normal basic structure formed by the mucosa and the submucosa layer, the smooth muscle layers, and the serosa layer.

### Effects of 28-day repeated oral administration of ciguatoxin-related compounds on food consumption, feces, and urine parameters

As shown in Table 7, none of the ciguatera toxins evaluated over the 28-day treatment period altered body weight or feces production, however urine production in mice treated with different doses of the toxins decreased as summarized in the same table. Therefore, urine parameters were evaluated after free catch collection of the samples during the 24-h period before euthanasia. Urine parameters analyzed in control and treated mice included color, clarity, specific gravity, protein, glucose, ketones, blood/hemoglobin, bilirubin, and urobilinogen are summarized in Supplementary Table 2.

**Table 7:** Food consumption, feces and urine production during the 24 h prior to sacrifice, in control mice and in animals treated orally with different doses of CTX3C, gambierone, MTX3 and MTX1 during 28 days

Control (*n* = 7)			
Food consumption (g)	5.3 ± 0.7		
Feces (g)	1.7 ± 0.3		
Urine (ml)	2.9 ± 0.9		
CTX3C (*n* = 3)	10 ng/kg	32 ng/kg	100 ng/kg
Food consumption (g)	3.9 ± 0.6	5 ± 0.9	4.7 ± 0.2
Feces (g)	1.6 ± 0.2	1.4 ± 0.4	1.3 ± 0.1
Urine (ml)	1.8 ± 0.4	1 ± 0.02	1.1 ± 0.5
Gambierone (*n* = 3)	172 ng/kg	500 ng/kg	1760 ng/kg
Food consumption (g)	2.9 ± 1.2	5.4 ± 0.6	5.2 ± 0.3
Feces (g)	1 ± 0.5	1.4 ± 0.4	1.6 ± 0.2
Urine (ml)	4.5 ± 1.6	1.3 ± 0.7	1.3 ± 0.2
MTX3	500 ng/kg (*n* = 2)	1760 ng/kg (*n* = 3)	
Food consumption (g)	3.8 ± 0.4	3.5 ± 0.3	
Feces (g)	1.2 ± 0.4	0.8 ± 1.4	
Urine (ml)	0.5 ± 0.3	2.2 ± 1.5	
MTX1	800 ng/kg (*n* = 3)	2560 ng/kg (*n* = 3; 1 surviving mouse)	5000 (*n* = 5), no surviving animals
Food consumption (g)	5.8 ± 0.8	5.2	
Feces (g)	1.5 ± 0.1	1.5	
Urine (ml)	1 ± 0.3	0.2	

Values are expressed as mean ± SEM

Interestingly, mice treated with CTX3C, at doses of 32 and 100 ng/kg had a slight decrease in urine production during the 24-h period after the last toxin dose with a urine volume of 1 ± 0.02 mL and 1.1 ± 0.5 mL, respectively (*n* = 3) versus the amount of urine collected from control animals during the same period which was 2.9 ± 0.9 mL in seven untreated animals, although these results did not reach statistically significant differences. Remarkably, urine color darkened after increasing the CTX3C dose, changing from pale yellow in control mice to dark yellow in mice treated with 100 ng/kg of CTX3C. Urine turbidity was also higher in mice treated with CTX3C at doses of 32 and 100 ng/kg. Besides, urine-specific gravity increased in animals treated with the highest toxin doses, in agreement with the increment in urine glucose levels. Furthermore, moderate urobilinogenuria was also present in the animals treated with the highest doses of CTX3C. Similarly, mice treated with gambierone at the dose of 1760 ng/kg had a remarkable decrease in urine production during the 24-h period after the last toxin dose and reached 1.3 ± 0.2 mL (*n* = 3). Urine color was darker, and turbidity and urine-specific gravity increased in gambierone-treated mice. Furthermore, there was a steep increase in the urine urobilinogen levels even at the lowest gambierone dose (170 ng/kg). The same effects were found in mice treated with MTX1, although in this case a smaller number of samples were obtained since one animal died overnight during the 24-h observation period.

## Discussion

Ciguatoxins are marine biotoxins involved in CFP, however other compounds produced by the marine phytoplankton also play an important role in this intoxication. So far, the available in vivo toxicity studies with the ciguatoxins and their related compounds causative of CFP, were mostly acute toxicity studies administering the toxins by the i.p. route (Hirama et al. 2001; Lewis and Hoy 1993; Murray et al. 2021; Vernoux and Lewis 1997; WHO 2020). Regarding the toxins belonging to the ciguatoxin group, the most potent analogue was P-CTX1B, with a mean i.p. LD_50_ of 0.32 μg/kg (WHO 2020) after i.p. administration of the toxin and the more potent analogue at the cellular level (Raposo-Garcia et al. 2022). Based on the available reports, CTX3C acute i.p. toxicity has also been established for mice at 1.6 μg/kg bw (Hirama et al. 2001; WHO 2020). However, in vivo toxicological data for many compounds presumably involved in CFP are still lacking due to their scarce commercial availability and the large number of existing analogues, a fact that led EFSA to indicate the need to perform more in vivo toxicity studies to settle the levels of these toxins allowed in food (EFSA 2010). The acute in vivo toxicity data for CTXs obtained so far indicated that the i.p. and oral routes had a similar LD_50_, suggesting a very high oral uptake of CTX (Bottein et al. 2011). Regarding gambierone, its i.p. acute toxicity in mice has been recently studied in a 14-day i.p. exposure establishing an acute LD_50_ of 2.4 mg/kg bw (Murray et al. 2021), while the LD_50_ for MTX3 in the same conditions was between 20 and 38 mg/kg, therefore, indicating a tenfold higher toxicity of gambierone versus MTX3 (Murray et al. 2020).

So far, the in vivo toxicity data for MTX1 is controversial since some reports had shown an acute LD_50_ by the i.p. route of 0.05 μg/kg, (Murata et al. 1993), however other reports using maitotoxin extracted from *Gambierdiscus toxicus* confirmed that i.p. injection of 200 and 400 ng/kg of MTX1 lead to ascites and gastrointestinal alterations without causing animal death (Terao et al. 1988). In contrast, a much lower toxicity of MTX1 in algal extracts has been recently described (Munday et al. 2017). Noteworthy, algal extracts containing only MTX1 and MTX3 were 100 times more toxic by the i.p. than by the oral route (Munday et al. 2017). Therefore, the in vivo data presented in this work that lead to obtain an i.p. LD_50_ for MTX1 of 1107 ng/kg remarks the need to reevaluate the effect of these compounds following internationality adopted guidelines. A summary of the wide range of LD_50_ for MTX1 proposed by different authors is reflected in Table 7 as extracted from (Munday 2014). One of the problems associated with establishing a reliable LD_50_ for MTX1 is the ambiguity of the toxicity terms previously reported (Munday 2014). Some of the early values reported with algal extracts and toxic fractions do not provide information about purity and presence of other toxic contaminants, hence these values should not be taken into account (Table 8).

**Table 8:** Diversity in the range of lethality doses described for purified MTX1 and algal extracts containing the toxic fraction

Route of administration/toxin origin	LD_50_ (ng/kg)	Animal model	References
I.p	50	mice	Murata et al. (1993)
I.p	130	mice	Yokoyama et al. (1988)
I.p	150	mice	Munday et al. (2017), Reyes et al. (2014)
I.p. (algal extracts)	160–1260	mice	Munday et al. (2017), Reyes et al. (2014)
I.p. (toxic fraction)	200,000	mice	Legrand and Bagnis (1984)
Oral (algal extracts)	16,000–100,000	mice	Munday et al. (2017)

To date, no additional reports on the chronic toxicity of any of these toxins have been published despite the worldwide increasing incidence of CFP. Nowadays it is known than more of 400 fish species can contain toxins involved in ciguatera (Canals et al. 2021) and ciguatera fish poisoning is considered an emerging food borne illness worldwide (WHO 2020) representing both environmental and public health challenges (Loeffler et al. 2021). During the last decade, the incidence of ciguatera in European coasts has increased progressively with ciguatoxins or their vectors been reported in France, Portugal and Spain (Bresnan et al. 2021; Canals et al. 2021; Costa et al. 2018; de Haro et al. 2021; Rossignoli et al. 2020). In a recent report from EFSA (Canals et al. 2021), both autochthonous and imported fish have been involved in ciguatera fish poisoning in Spain, Germany, France, Austria, Portugal and Switzerland, a fact that reveals the need to establish regulatory levels for ciguatera as well as to include these toxins on the surveillance controls of marine toxins (Katikou 2021).

The lack of data on the chronic oral toxicity of all of the compounds causative of CFP as well as the opinions of EFSA on the need to evaluate the toxicity of ciguatoxin-related compounds (Canals et al. 2021; EFSA 2010) prompted us to study their in vivo effects. Indeed the American Food and Drug Administration (FDA) has implemented a maximum limit of 0.01 mg/kg fish of P-CTX1 or 0.1 mg/kg fish of C-CTX1 before taken legal action to remove fish from the market (FDA 2021). Usually ciguatera fish poisoning has been associated with the ingestion of fishes such us tuna, red snapper, mackerel, barracuda, warsaw grouper, hotfish and amberjack (WHO 2020). While not regulatory levels in fishery products for the compounds belonging to the ciguatoxin group have been yet adopted in the European Union, the current legislation does not allow fish containing ciguatoxins to be commercialized (EC 2019) (EC 2019; Friedman et al. 2008). In our study, daily oral administration of CTX3C (10–102 ng/kg) to mice during a month altered urine parameters but did not cause observable symptoms or lethality.

Furthermore, acute exposure of mice to MTX1 or CTX3C by the i.p. route revealed a NOAEL for MTX1 below 200 ng/kg after a 96-h observation period while for the acute oral administration of MTX1 no symptoms or biochemical alterations were observed at doses of 800 ng/kg thus confirming the lower effect of MTX1 by the oral route, as previously described (Munday et al. 2017). For CTX3C, no symptoms or deaths were observed at the higher dose evaluated, 1050 ng/kg of CTX3C, a fact that is in agreement with its i.p. LD_50_ ranging from 1200 to 2500 ng/kg (Dechraoui et al. 1999; Yogi et al. 2014).

To complete the data available as well as to obtain an approach to the repeated oral exposure toxicity of MTX1 and CTX3C daily dosage of mice with the toxins was performed. In this context, the data presented in this report constitute the first approach for the evaluation of their toxicity after daily ciguatoxin ingestion. The results presented in this work indicated that CTX3C at doses of 102 ng/kg and gambierone administered daily at 1700 ng/kg did not cause animal death but altered blood potassium levels. While for MTX1 doses of 800 ng/kg, for 28 days did not cause animal death nor any macroscopic organ alterations at necropsy, however doses of 2560 ng/kg and 5000 ng/kg of the toxin caused animal death and difficulty to obtain samples for biochemical analysis. These difficulties in obtaining samples for biochemical analysis due to the animal treatment, sudden dead and the poor condition of some of the animals treated before sacrifice do not allow these parameters to be compared.

The approach to obtain data about the acute i.p. toxicity of MTX1 yielded an estimated LD_50_ of 1107 ng/kg after a 96-h observation period, a result that should be taken into account in view of the huge range of LD_50_ doses reported so far for MTX1 which vary from 50 to 200,000 ng/kg (Munday et al. 2017; Murata et al. 1994; Reyes et al. 2014; Yokoyama et al. 1988). In agreement with previous studies on the i.p. toxicity of MTX1 (Murata et al. 1993), the lowest i.p. dose employed in this work, 0.2 μg/kg, already caused lethargy, piloerection (1/3 mice), and stretching (two out of three animals), however, at this dose, neither alterations in ALT, AST, CK and LDH parameters nor in the electrolyte balance were observed. When mice were fed with a single MTX1 dose of 800 ng/kg no mortality was observed and animals showed only transitory minor symptoms like lethargy and piloerection, but not biochemical alterations in blood parameters were detected. In the case of a single oral dose of CTX3C of either 330 or 1050 ng/kg, animals did not have symptoms or alterations in the blood parameters during the 96-h observation period. In the case of MTX1, significant macroscopic alterations in the digestive tract produced by i.p. administration of the toxin at doses from 1000 ng/kg and higher is in accordance with the increase in mortality rate. However, lower doses studied did not elicited neither death nor macroscopic alterations in animals which indicates that the toxicity of MTX1 is lower than that previously described (Murata et al. 1993).

Important in vivo disturbances by the CTX-related compounds employed in this work occurred during the repeated oral administration of these compounds. We demonstrated here that, after daily feeding of mice with MTX1 for 28 days, at a dose of 800 ng/kg only affected urine parameters. However, daily oral dosage of mice with MTX1 at 5000 ng/kg led to a mortality rate of 100% with survival times between 24 h and 15 days highlighting the need to pursue detailed studies about the chronic effects of these compounds.

The changes in urine parameters caused by ciguatoxins may suggest that the glomerular filtration barrier could be damaged as previously described for other sodium channel modulators (Boente-Juncal et al. 2020). These data are in agreement with the persistent oliguria described previously in rats after single i.p. or oral administration of P-CTX1 at 2600 ng/kg (Bottein et al. 2011). A fact that could arise from the demonstrated presence of active ciguatoxins in urine and feces during 4 days after exposure (Bottein et al. 2011) attributed to a poor elimination of the toxin in urine or to the reabsorption of the toxin in the urinary system. The fact that the same effect was caused by CTX3C at doses of 330 and 1050 ng/kg remarks the need to revaluate the toxicity of the ciguatoxin group of compounds since the effect of CTX3C was evident at doses similar to that used for P-CTX1. In mice, no quantitative assessment of the CTXs bioavailability has been described, however, conducted LD_50_ studies (i.p. and oral) using semi purified toxins (> 85 percent) on male Institute of Cancer Research (ICR) mice (4 weeks old), suggested a very high oral uptake with identical LD_50_ values and almost similar signs of intoxications for the two routes of administration (Ito et al. 1996; Lehane and Lewis 2000; Lewis and Hoy 1993; Lewis et al. 1993).

Altogether, the new findings described in the present study together with the increase of CFP-reported cases in Europe, raise the need to perform additional studies on the chronic oral in vivo toxicity of this marine group of toxins to prevent human illness and accurately regulate the food levels of these emerging marine toxins.

## Conclusion

The toxicity of ciguatoxin-related compounds needs to be reevaluated since there is a wide range of lethal doses in the literature for all these compounds.

The toxicity of MTX1 historically considered one of the most toxic natural compounds need to be reevaluated since this work evidence that much higher MTX1 doses did not elicit neither animal dead nor organs alterations.

CTX3C, Gambierone and MTX3 did not cause death after a 28-day oral administration period.

The results presented here evidence the need to evaluate the toxicity of environmental pollutants using reference materials and harmonized validated protocols.

## Supplementary Information

Below is the link to the electronic supplementary material.

Supplementary file1 (DOCX 6933 KB)

Supplementary file2 (PDF 68 KB)

Supplementary file3 (PDF 96 KB)

## Abbreviations

CFP: Ciguatera fish poisoning
CTX: Ciguatoxins
HAB: Harmful algal bloom
MTX: Maitotoxin
LD_50_: Lethal dose 50
i.p.: Intraperitoneal
bw: Body weight
H&E: Haematoxilin-eosin
FDA: American Food and Drug Administration
EFSA: European Food Safety Authority
