# Supplementary material for: In vivo subchronic effects of ciguatoxin-related compounds, reevaluation of their toxicity
Source: Arch Toxicol. 2022 Jun 3;96(9):2621–38. doi: 10.1007/s00204-022-03315-0 (PMC9325831; doi:10.1007/s00204-022-03315-0)
Supplement: Supplementary file 1 — Supplementary file1 (DOCX 6933 KB) [file 204_2022_3315_MOESM1_ESM.docx]

***In vivo* subchronic effects of ciguatoxin-related compounds, reevaluation of their toxicity**

*Sandra Raposo-García^1^, Andrea Boente-Juncal^1^, Mercedes Rodriguez-Vieytes^2^, Mercedes Camiña^2^, Celia Costas^1^, Alejandro Cao^1^, M. Carmen Louzao, Manuel Cifuentes^3^, Carmen Vale*, Luis M. Botana**

* Correspondence: luis.botana@usc.es, mdelcarmen.vale@usc.es; Tel.: +34-982822233 (L.M.B).

^1^Departamento de Farmacología, Farmacia y Tecnología Farmacéutica, Facultad de Veterinaria, Universidad de Santiago de Compostela, Campus Universitario s/n, 27002, Lugo, España.

^2^Departamento de Fisiología, Facultad de Veterinaria, Universidad de Santiago de Compostela, Campus Universitario s/n, 27002, Lugo, España.

^3^Departamento de Anatomía, Producción Animal y Ciencias Clínicas Veterinarias. Facultad de Veterinaria, Universidad de Santiago de Compostela, Campus Universitario s/n, 27002, Lugo, España.

[Sandra.raposo.garcia@usc.es](mailto:Sandra.raposo.garcia@usc.es); [andrea.boente.juncal@usc.es](mailto:andrea.boente.juncal@usc.es); [mmercedes.rodriguez@usc.es](mailto:mmercedes.rodriguez@usc.es); [merchi.camina@usc.es](mailto:merchi.camina@usc.es); [celia.costas.sanchez@usc.es](mailto:celia.costas.sanchez@usc.es); [mcarmen.louzao@usc.es](mailto:mcarmen.louzao@usc.es); [m.cifuentes@usc.es](mailto:m.cifuentes@usc.es); [mdelcarmen.vale@usc.es](mailto:mdelcarmen.vale@usc.es); [luis.botana@usc.es](mailto:luis.botana@usc.es)


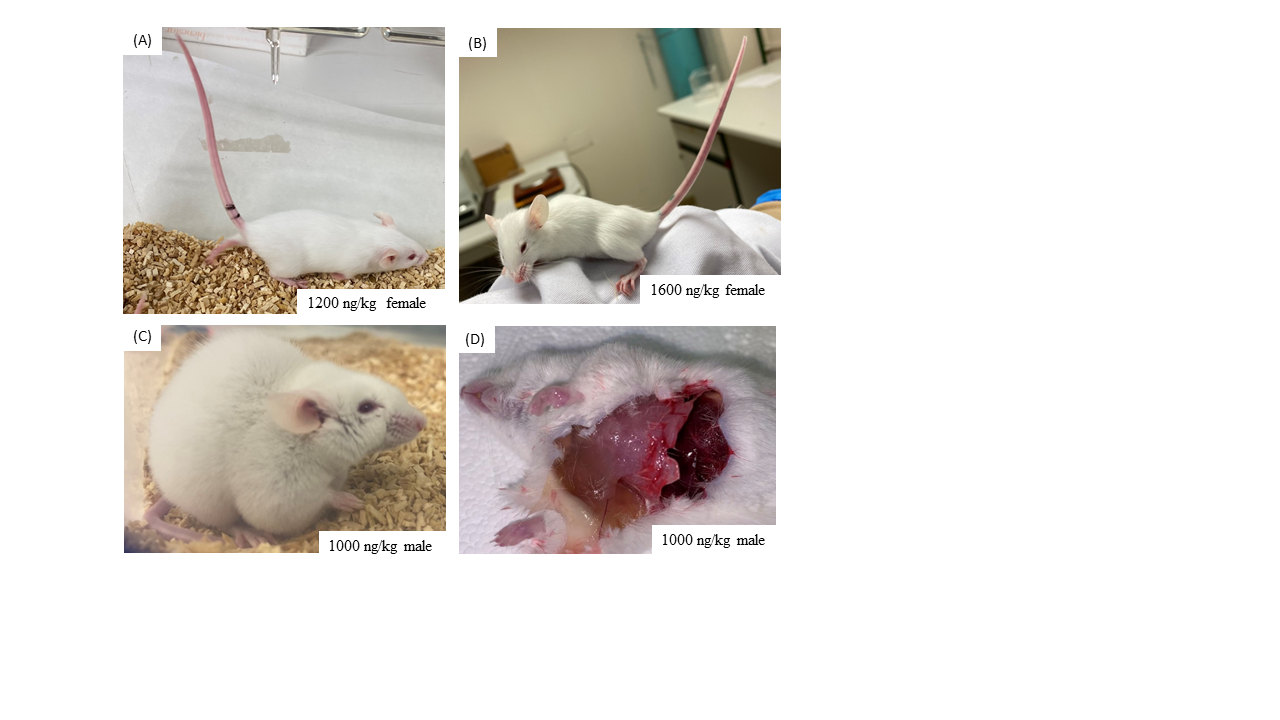


**Supplementary Figure 1.** Straub´s tail reaction after i.p. administration of different doses of MTX1 (A and B). Subcutaneous edema was obvious in a mouse treated with 1,000 ng/kg of MTX1 before (C) and after (D) euthanasia.


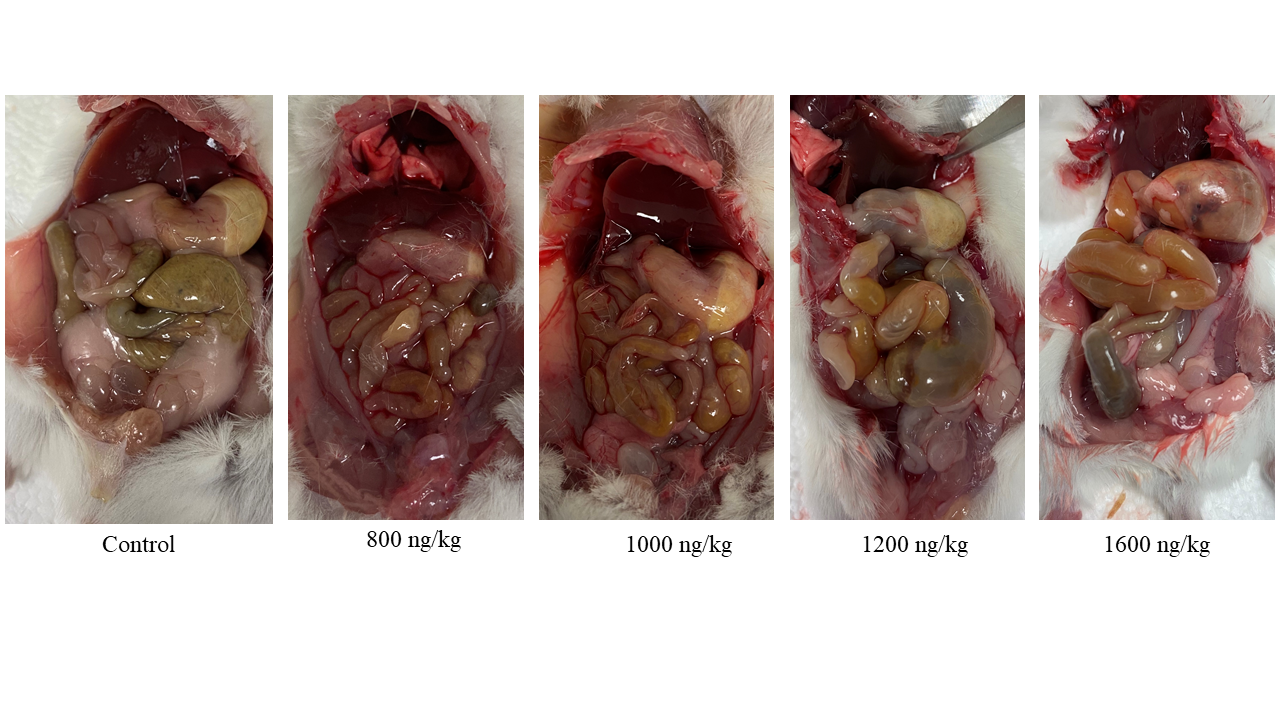


**Supplementary Figure 2.** Representative image of the abdominal tract of control mice and animals treated intraperitoneally with different MTX1 doses.


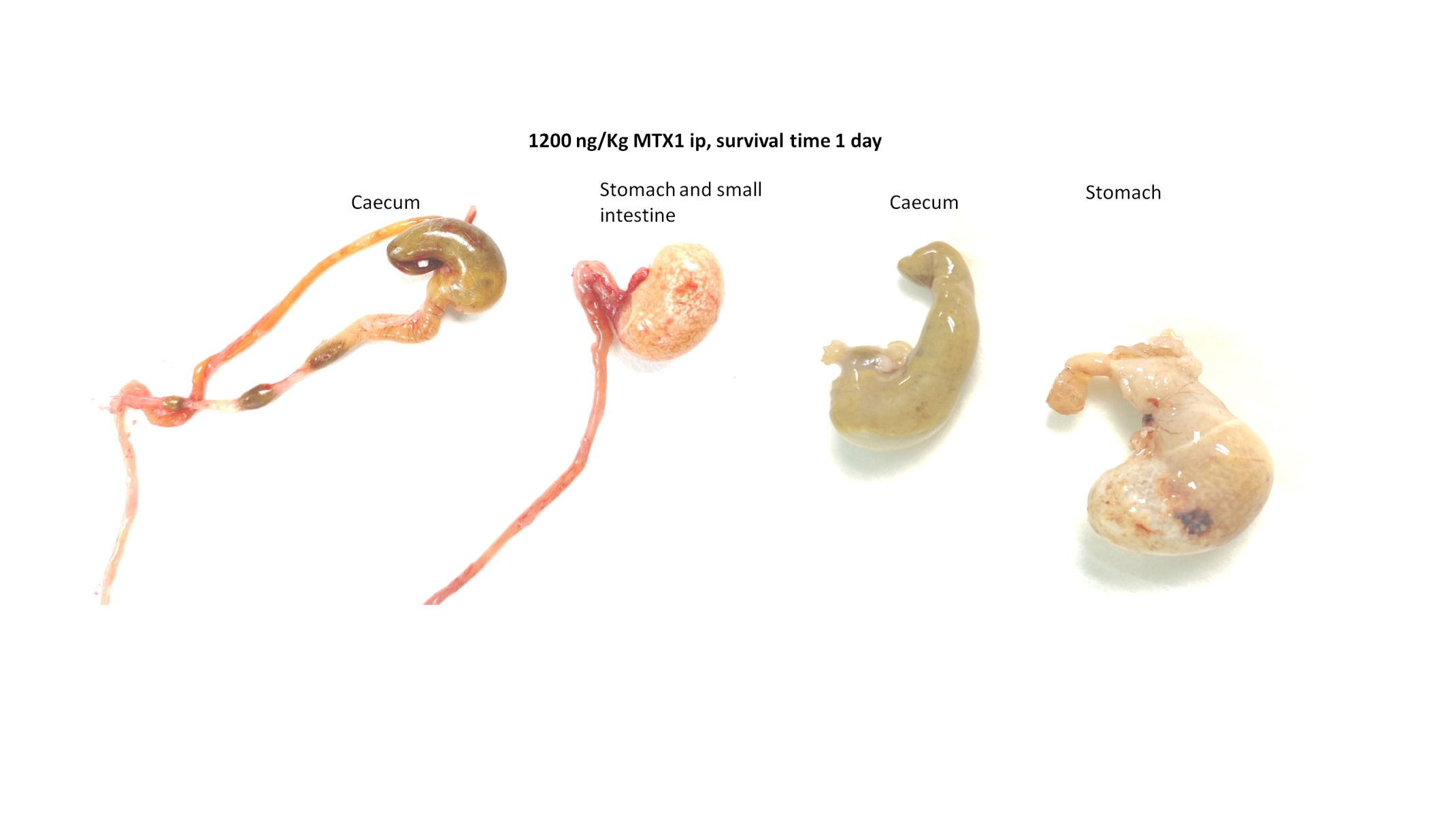


**Supplementary Figure 3.** Haemorrhagic zones present in the stomach of animal treated with 1,200 ng/Kg of MTX1. As shown in the right these animals have the intestines empty of solid content and the caecum full of gas.

**
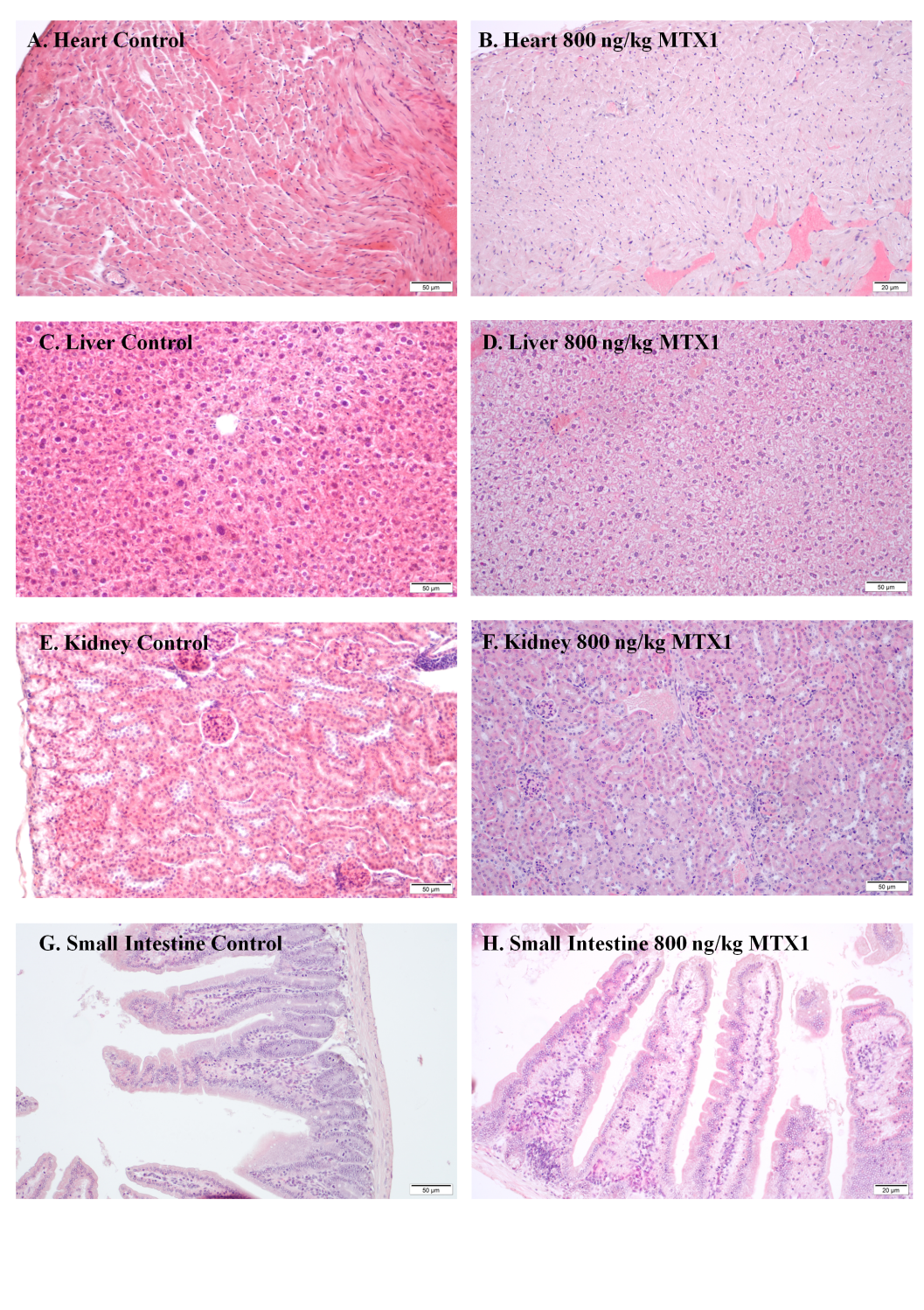
**

**Supplementary Figure 4**. Representative hematoxylin–eosin (H&E) staining of different organs of control mice (A, C, E, G) and mice treated with 800 ng/kg MTX1 in a single i.p. administration after a 96-hour observation period (B, D, F, H). Scale bar is 50 µm.

^^

**Supplementary Figure 5**. Metabolic cage parameters after acute intraperitoneal injection of CTX3C. A. Food consumption. B. Feces production. C. Urine production. *p < 0.05, **p < 0.01 vs control values. Data are expressed as mean ± SEM from 3 animals.

**Supplementary Table 1**. Repercussion of repeated oral administration of control diluent, CTX3C, gambierone, MTX3 and MTX1 on body weight for the 28-day treatment period. Values are expressed as mean ± SEM and the number of animals is represented in parenthesis.

|  | **Day 0** | **Day 7** | **Day 14** | **Day 21** | **Day 28** |
| --- | --- | --- | --- | --- | --- |
| **Body weight (g)** | **(n = 7)** | **(n = 7)** | **(n = 7)** | **(n = 7)** | **(n = 7)** |
| **Control** | 23.1 ± 1 | 24.6 ± 0.8 | 26.7 ± 0.6 | 27.7 ± 0.7 | 27.6 ± 0.7 |
| **CTX3C** | **(n = 3)** | **(n = 3)** | **(n = 3)** | **(n = 3)** | **(n = 3)** |
| 10 ng/kg | 26.5 ± 0.7 | 27.5 ± 0.4 | 27.6 ± 0.5 | 28.1 ± 1 | 28.40 ± 1.3 |
| 32 ng/ kg | 24.4 ± 0.7 | 25.8 ± 0.7 | 27.2 ± 1.3 | 28.3 ± 1.2 | 29.3 ± 1.4 |
| 102 ng/ kg | 26.3 ± 0.3 | 26.8 ± 1 | 26.9 ± 0.9 | 26.2 ± 0.74 | 26.1 ± 1.4 |
| **Gambierone** | **(n = 3)** | **(n = 3)** | **(n = 3)** | **(n = 3)** | **(n = 3)** |
| 172 ng/kg | 25.9 ± 0.4 | 26.6 ± 0.4 | 27.2 ± 0.2 | 27.9 ± 0.3 | 28.4 ± 1.4 |
| 550 ng/kg | 22.6 ± 1.1 | 23.8 ± 1.7 | 25.5 ± 2.2 | 26.8 ± 2.6 | 28.3 ± 2.8 |
| 1760 ng/kg | 26.4 ± 0.8 | 26.3 ± 0.9 | 25.8 ± 0.8 | 26.1 ± 0.4 | 25.7 ± 1.1 |
| **MTX3** |  |  |  |  |  |
| 550 ng/kg | 24.5 ± 1.5 (n = 2) | 24.2 ± 1.2 (n = 2) | 25 ± 1.9 (n = 2) | 25.1 ± 2.1 (n = 2) | 25.2 ± 2.1 (n = 2) |
| 1760 ng/kg | 24.7 ± 1.3 (n = 3) | 26.1 ± 1.6 (n = 3) | 26.8 ± 1.3 (n = 3) | 27.6 ± 1.7 (n = 3) | 27.7 ± 1.5 (n = 3) |
| **MTX1** |  |  |  |  |  |
| 800 ng/kg | 26 ± 1.7 (n = 3) | 26.4 ± 1.5 (n = 3) | 27.5 ± 1.8 (n = 3) | 28.6 ± 1.7 (n = 3) | 29.4 ± 2.2 (n = 3) |
| 2560 ng/kg | 24.2 ± 0.7 (n = 3) | 26.5 ± 0.3 (n = 3) | 25.8 ± 1.6 (n = 2) | 22.8 (n = 1) | 23.4 (n = 1) |
| 5000 ng/kg | 23.1 ± 1.3 (n = 5) | 22.4 ± 2 (n = 3) | 21.3 ± 2 (n = 3) |  |  |

**Supplementary Table 2.** Analytical results of urine after repeated oral administration of control diluent and CTX3C, gambierone, MTX1 and MTX3 to mice for 28 days. In the urinalysis parameters as urine color, turbidity, specific gravity, urine protein, glucose, ketones, blood/hemoglobin, bilirubin, and urobilinogen were considered. Results are expressed as mean ± SEM and the number of animals appeared in parenthesis. * n = 3 values except for maitotoxin-1 treated animals that died during the treatment.

| **Parameters** | **(7) Control** | **(3) 10 ng/kg CTX3C** | **(3) 32 ng/kg CTX3C** | **(3) 102 ng/kg CTX3C** | **(2) 170 ng/kg Gambierone** | **(3) 500 ng/kg Gambierone** | **(3) 1760 ng/kg Gambierone** | **(3) 500 ng/kg MTX3** | **(3) 1760 ng/kg MTX3** | **(3) 800 ng/kg MTX1** | **(1) 2600 ng/kg MTX1** |
| --- | --- | --- | --- | --- | --- | --- | --- | --- | --- | --- | --- |
| **Colour** | (5) Pale Yellow  (1) Amber  (1) Dark Yellow | (2) Pale Yellow  (1) Dark Yellow | (1) Amber  (2) Dark Yellow | (3) Dark Yellow | (2) Pale Yellow | (1) Pale Yellow  (2) Dark Yellow | (3) Dark Yellow | (2) Amber | (1) Amber  (2) Dark Yellow | (3) Pale Yellow | (1) Dark Yellow |
| **Turbididy** | (4) Clear  (2) Slightly cloudy  (1) Cloudy | (1) Clear  (2) Slightly cloudy | (1) Clear  (2) Cloudy | (3) Cloudy | (2) Clear | (1) Slightly  (2) Cloudy | (3) Cloudy | (1) Cloudy  (1) Very cloudy | (3) Very cloudy | (3) Cloudy | (1) Cloudy |
| **Specific Gravity** | (1) 1020  (1) 1030  (1) 1032  (3) >1050 | (1) 1046  (1) 1050  (1) >1050 | (3) >1050 | (3) >1050 | (1) 1020  (1) 1036 | (1) 1050  (2) > 1050 | (3) > 1050 | (1) > 1050  (1) 1044 | (1) 1016  (2) > 1050 | (3) > 1050 | (1) > 1050 |
| **Urine protein (g/l)** | 0.3 ± 0.3  (3) 0  (3) Trace  (1) 1 | 0 ± 0  (1) 0.3  (2) Trace | 1  (2) Trace  (1) 1 | 0.3 ± 0  (1) Trace  (3) 0.3 | 2.1 ± 1.5  (1) 0.3  (1) 1  (1) 5 | 0.65 ± 0.35  (1) 0.3  (1) 1 | :0.5 ± 0.2  (2) 0.3  (1) 1 | 0.3 ± 0  (2) 0.3 | 0.2 ± 0.1  (2) 0.3  (1) 0 | 0.3 ± 0  (1) Trace  (2) 0.3 | (1) 1 |
| **Glucose (mmol/l)** | 0.9 ± 0.6  (5) 0  (2) 3 | 1 ± 1  (2) 0  (1) 3 | 5.8 ± 2.8  (4) 3  (1) 17 | 3 ± 0  (4) 3 | 0 ± 0  (2) 3 | 0 ± 0  (2) 0 | 0 ± 0  (3) 0 | 1.5 ± 1.5  (1) 0  (1) 3 | 1 ± 1  (1) 0  (2) 3 | 1 ± 1  (1) 0  (2) 3 | (1) 0 |
| **Ketones (mmol/l)** | 0.3 ± 0.2  (3) 0  (3) 1.5 | 0.3 ± 0.3  (4) 0  (1) 1.5 | 1.2 ± 0.3  (1) 0  (4) 1.5 | 0.4 ± 0.4  (3) 0  (1) 1.5 | 0.8 ± 0.8  (1) 0  (1) 1.5 | 0 ± 0  (2) 0 | 1 ± 0.5  (1) 0  (2) 1.5 | 0 ± 0  (2) 0 | 1 ± 0.5  (1) 0  (2) 1.5 | 0.5 ± 0.5  (2) 0  (1) 1.5 | (1) 5 |
| **Blood/Haemoglobin (ery/µl)** | 0 ± 0  (7) 0 | 0 ± 0  (5) 0 | 12 ± 9.7  (3) 0  (1) 10  (1) 50 | 2.5 ± 2.5  (3) 0  (1) 10 | 0 ± 0  (2) 0 | 5 ± 5  (1) 0  (1) 10 | 0 ± 0  (3) 0 | 10 ± 10  (1) 0  (1) 10 | 8.3 ± 8.3  (2) 0  (1) 25 | 0 ± 0  (3) 0 | (1) 0 |
| **Bilirubin (µmol/l)** | 24 ± 14  (4) 0  (1) 17  (1) 50  (1) 100 | 10.2 ± 4.2  (2) 0  (3) 17 | 43.4 ± 6.6*  (1) 17  (4) 50 | 25.3 ± 8.3  (3) 17  (1) 50 | 17 ± 0  (2) 17 | 50 ± 0*  (2) 50 | 11.3 ± 5.7  (1) 0  (2) 17 | 8.5 ± 8.5  (1) 0  (1) 17 | 17 ± 0  (3) 17 | 83 ± 16.7  (1) 50  (2) 100 | (1) 50 |
| **Urobilinogen (µmol/l)** | 0 ± 0  (7) 0 | 10.2 ± 4.2  (2) 0  (3) 17 | 6.8 ± 4.2  (3) 0  (2) 17 | 0 ± 0  (4) 0 | 8.5 ± 8.5  (1) 0  (1) 17 | 8.5 ± 8.5  (1) 0  (1) 17 | 11.3 ± 5.7  (1) 0  (2) 17 | 8.5 ± 8.5  (1) 0  (1) 17 | 0  (3) 0 | 5.7 ± 5.7  (2) 0  (1) 17 | (1) 70 |
