# Supplementary material for: In vivo subchronic effects of ciguatoxin-related compounds, reevaluation of their toxicity
Source: Arch Toxicol. 2022 Jun 3;96(9):2621–38. doi: 10.1007/s00204-022-03315-0 (PMC9325831; doi:10.1007/s00204-022-03315-0)
Supplement: Supplementary file 2 — Supplementary file2 (PDF 68 KB) [file 204_2022_3315_MOESM2_ESM.pdf]

SAFETY DATA SHEET

According to OSHA HazCom 2012
Revision Date 06-Oct-2020
Version 1

1. IDENTIFICATION OF THE SUBSTANCE/PREPARATION AND OF THE COMPANY/UNDERTAKING

Product identifier
Product Name Maitotoxin

Other means of identification
Product Code 131-19011
UN/ID No UN3462

Recommended use of the chemical and restrictions on use
Recommended Use No information available.
Uses advised against No information available

Details of the supplier of the safety data sheet
Distributor
FUJIFILM Wako Pure Chemical Corporation . 1-2 Doshomachi 3-Chome, Chuo-ku, Osaka 540-8605, Japan Phone: +81 (0)6-6203-3741 Fax: +81 (0)6-6201-5964
FUJIFILM Wako Chemicals U.S.A., Inc. 1600 Bellwood Road, Richmond, VA 23237-1326, U.S.A. Phone: +1 (0)804-271-7677 Fax: +1 (0)804-271-7791
FUJIFILM Wako Chemicals GmbH Fuggerstrasse 12, D-41468 Neuss, Germany Phone: +49 (0)2131-311 158 Fax: +49 (0)2131-311 100

2. HAZARDS IDENTIFICATION

GHS classification
Classification of the substance or mixture

**Acute toxicity - Oral**	Category 1
**Specific target organ toxicity (single exposure)**	Category 1

Pictograms

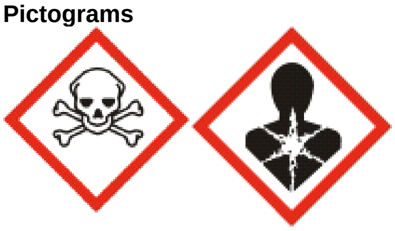

Signal word Danger

Hazard statements
H300 - Fatal if swallowed
H370 - Causes damage to organs

Precautionary statements-(Prevention)
Wash face, hands and any exposed skin thoroughly after handling Do not eat, drink or smoke when using this product Do not breathe dust/fume/gas/mist/vapors/spray
Precautionary statements-(Response)
IF exposed: Call a POISON CENTER or doctor/physician
IF SWALLOWED: Immediately call a POISON CENTER or doctor/physician Rinse mouth.
Precautionary statements-(Storage)
Store locked up.

Precautionary statements-(Disposal)
Dispose of contents/container to an approved waste disposal plant

Others
Other hazards Not available

3. COMPOSITION/INFORMATION ON INGREDIENTS

Single Substance or Mixture Substance

Formula C164H256Na2O68S2

Chemical Name	Molecular weight	CAS RN	Weight-%
Maitotoxin	3425.86	59392-53-9	=<100

Impurities and/or Additives： Not applicable

4. FIRST AID MEASURES

First aid measures

Eye contact Rinse thoroughly with plenty of water for at least 15 minutes, lifting lower and upper eyelids. Consult a physician.

Skin contact Wash skin with soap and water.

Inhalation Remove to fresh air.

Ingestion Rinse mouth. Never give anything by mouth to an unconscious person. Call a physician or poison control center immediately. Do not induce vomiting without medical advice.

Most important symptoms and effects, both acute and delayed

Symptoms No information available.

Indication of any immediate medical attention and special treatment needed

Note to physicians Treat symptomatically.

5. FIRE-FIGHTING MEASURES

Suitable Extinguishing media
Use extinguishing measures that are appropriate to local circumstances and the surrounding environment.

Unsuitable Extinguishing media Caution: Use of water spray when fighting fire may be inefficient.

Specific hazards arising from the chemical
No information available.

Explosion data
Sensitivity to Mechanical Impact none.
Sensitivity to Static Discharge none.

Protective equipment and precautions for firefighters
As in any fire, wear self-contained breathing apparatus pressure-demand, MSHA/NIOSH (approved or equivalent) and full protective gear.

6. ACCIDENTAL RELEASE MEASURES

Personal precautions, protective equipment and emergency procedures

**Personal precautions, protective equipment and emergency procedures**	Ensure adequate ventilation, especially in confined areas.

Environmental precautions

**Environmental precautions**	See Section 12 for additional ecological information.

Methods and material for containment and cleaning up

**Methods and material for containment and cleaning up**	Prevent further leakage or spillage if safe to do so.
**Methods for cleaning up**	Pick up and transfer to properly labeled containers.

7. HANDLING AND STORAGE

Precautions for safe handling

Technical measures	Avoid contact with strong oxidizing agents.
**Protective measures**	Handle in accordance with good industrial hygiene and safety practice.

Conditions for safe storage, including any incompatibilities

**Storage conditions**	Container protected from light, and store tightly closed in freezer (-20°C).
**Packaging materials**	Glass.
**Incompatible materials**	Strong oxidizing agents.

8. EXPOSURE CONTROLS/PERSONAL PROTECTION

Engineering controls

In case of indoor workplace, seal the source or use a local exhaust system. Provide the safety shower facility, and hand- and eye-wash facility. And display their position clearly.

**Exposure limits**	This product, as supplied, does not contain any hazardous materials with occupational exposure limits established by the region specific regulatory bodies.

Personal protective equipment

**Respiratory protection**	Dust mask
**Hand protection**	Protection gloves
**Eye protection**	protective eyeglasses or chemical safety goggles
Skin and body protection	Long-sleeved work clothes

General hygiene considerations

Handle in accordance with good industrial hygiene and safety practice.

9. PHYSICAL AND CHEMICAL PROPERTIES

**Form**	
**Appearance**	film
**Odor**	No data available
**pH**	No data available
**Melting point/freezing point**	No data available
**Boiling point, initial boiling point and boiling range**	No data available
**Flash point**	No data available
**Evaporation rate:**	No data available
**Flammability (solid, gas):**	No data available
**Upper/lower flammability or explosive limits**	
**Upper**：	No data available
**Lower**：	No data available

**Vapour pressure**	No data available
**Vapour density**	No data available
**Specific Gravity / Relative density**	No data available
**Solubilities**	methanol , DMSO : soluble .
**n-Octanol/water partition coefficient:(log Pow)**	No data available
**Auto-ignition temperature:**	No data available
**Decomposition temperature:**	No data available
**Viscosity (coefficient of viscosity)**	No data available
**Dynamic viscosity**	No data available

10. STABILITY AND REACTIVITY

Stability

Chemical stability May be altered by light.
Reactivity No data available

Hazardous reactions
None under normal processing

Conditions to avoid
Extremes of temperature and direct sunlight

Incompatible materials
Strong oxidizing agents

Hazardous decomposition products
Carbon monooxide (CO), Carbon dioxide (CO2), Sulfur oxides (SOx)

11. TOXICOLOGICAL INFORMATION

**Acute toxicity**	No data available
**Skin irritation/corrosion**	No data available
**Serious eye damage/ irritation**	No data available
**Respiratory or skin sensitization**	No data available
**Reproductive cell mutagenicity**	No data available
**Carcinogenicity**	No data available
**Reproductive toxicity**	No data available
**STOT-single exposure**	No data available
**STOT-repeated exposure**	No data available
**Aspiration hazard**	No data available

12. ECOLOGICAL INFORMATION

Ecotoxicity
No information available

Persistence and degradability
No information available

Bioaccumulative potential
No information available

Mobility in soil No information available
Other Data No information available

13. DISPOSAL CONSIDERATIONS

Waste treatment methods

**Disposal of wastes**	Disposal should be in accordance with applicable regional, national and local laws and regulations.
**Precautionary including method of disposing contaminated packaging**	Disposal should be in accordance with applicable regional, national and local laws and regulations.

14. TRANSPORT INFORMATION

DOT

**UN/ID No**	UN3462
**Proper shipping name:**	Toxins, extracted from living sources, solid, n.o.s. (Maitotoxin)
**UN classfication**	6.1
**Subsidiary hazard class**	
**Packing group**	I
**Marine pollutant**	Not applicable

IATA

**UN/ID No**	UN3462
**Proper shipping name:**	Toxins, extracted from living sources, solid, n.o.s. (Maitotoxin)
**UN classfication**	6.1
**Subsidiary hazard class**	
**Packing group**	I
**Environmentally Hazardous Substance**	Not applicable

IMDG

**UN/ID No**	UN3462
**Proper shipping name:**	Toxins, extracted from living sources, solid, n.o.s. (Maitotoxin)
**UN classfication**	6.1
**Subsidiary hazard class**	
**Packing group**	I
**Marine pollutant (Sea)**	Not applicable

15. REGULATORY INFORMATION

International Inventories

**TSCA**	-
**DSL**	-
**NDSL**	-

Legend:

TSCA - United States Toxic Substances Control Act Section 8(b) Inventory
DSL/NDSL - Canadian Domestic Substances List/Non-Domestic Substances List

US Federal Regulations

SARA 313

Section 313 of Title III of the Superfund Amendments and Reauthorization Act of 1986 (SARA). This product does not contain any chemicals which are subject to the reporting requirements of the Act and Title 40 of the Code of Federal Regulations, Part 372

SARA 311/312 Hazard Categories

**Acute health hazard**	No
**Chronic Health Hazard**	No
**Fire hazard**	No
**Sudden release of pressure hazard**	No
**Reactive Hazard**	No

CWA (Clean Water Act)

This product does not contain any substances regulated as pollutants pursuant to the Clean Water Act (40 CFR 122.21 and 40 CFR 122.42)

CERCLA

This material, as supplied, does not contain any substances regulated as hazardous substances under the Comprehensive Environmental Response Compensation and Liability Act (CERCLA) (40 CFR 302) or the Superfund Amendments and Reauthorization Act (SARA) (40 CFR 355). There may be specific reporting requirements at the local, regional, or state level pertaining to releases of this material

US State Regulations

California Proposition 65

This product does not contain any chemicals regulated by Proposition 65

U.S. State Right-to-Know Regulations

U.S. EPA Label Information

**EPA Pesticide Registration Number**	Not applicable

16. OTHER INFORMATION

**Issue Date**	05-Oct-2020
**Revision Date**	06-Oct-2020

Revision Note

No information available

Disclaimer

The information provided in this Material Safety Data Sheet is correct to the best of our knowledge, information and belief at the date of its publication. The information given is designed only as a guidance for safe handling, use, processing, storage, transportation, disposal and release and is not to be considered a warranty or quality specification. The information relates only to the specific material designated and may not be valid for such material used in combination with any other materials or in any process, unless specified in the text.

End of Safety Data Sheet

Suitable extinguishing media

Water spray (fog), Carbon dioxide (CO2), Foam, Extinguishing powder, Sand

Unsuitable extinguishing media

No information available

Special extinguishing method

No information available

Specific hazards arising from the chemical product

Thermal decomposition can lead to release of irritating and toxic gases and vapors.

Special protective actions for fire-fighters

Use personal protective equipment as required. Firefighters should wear self-contained breathing apparatus and full firefighting turnout gear.
