# Supplementary material for: In vivo subchronic effects of ciguatoxin-related compounds, reevaluation of their toxicity
Source: Arch Toxicol. 2022 Jun 3;96(9):2621–38. doi: 10.1007/s00204-022-03315-0 (PMC9325831; doi:10.1007/s00204-022-03315-0)
Supplement: Supplementary file 3 — Supplementary file3 (PDF 96 KB) [file 204_2022_3315_MOESM3_ESM.pdf]

According to OSHA HazCom 2012
Revision Date 23-Oct-2019
Version 2.01

SAFETY DATA SHEET

Details of the supplier of the safety data sheet
Distributor
FUJIFILM Wako Pure Chemical Corporation . 1-2 Doshomachi 3-Chome, Chuo-ku, Osaka 540-8605, Japan Phone: +81 (0)6-6203-3741 Fax: +81 (0)6-6201-5964
FUJIFILM Wako Chemicals USA, Inc. 1600 Bellwood Road, Richmond, VA 23237-1326, U.S.A. Phone: +1 (0)804-271-7677 Fax: +1 (0)804-271-7791
FUJIFILM Wako Chemicals GmbH Fuggerstrasse 12, D-41468 Neuss, Germany Phone: +49 (0)2131-311 158 Fax: +49 (0)2131-311 100

2. HAZARDS IDENTIFICATION

1. IDENTIFICATION OF THE SUBSTANCE/PREPARATION AND OF THE COMPANY/UNDERTAKING

GHS classification
Classification of the substance or mixture

**Acute toxicity - Oral**	Category 1
**Specific target organ toxicity (single exposure)**	Category 1

Category 1 nervous system

Pictograms

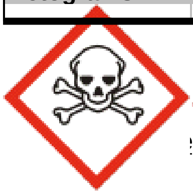
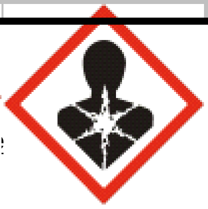

Signal word Danger

Other means of identification CiguatoxinCTX 3C
Product Code 030-21581
UN/ID No UN3462

Hazard statements
H300 - Fatal if swallowed
H370 - Causes damage to the following organs: nervous system

Precautionary statements-(Prevention)
Wash face, hands and any exposed skin thoroughly after handling Do not eat, drink or smoke when using this product Do not breathe dust/fume/gas/mist/vapors/spray
Precautionary statements-(Response)
IF exposed: Call a POISON CENTER or doctor/physician
IF SWALLOWED: Immediately call a POISON CENTER or doctor/physician Rinse mouth.
Precautionary statements-(Storage)

Recommended use of the chemical and restrictions on use

**Recommended Use**	No information available.
**Uses advised against**	No information available

Store locked up.

Precautionary statements-(Disposal)

Dispose of contents/container to an approved waste disposal plant

Others

Other hazards Not available

3. COMPOSITION/INFORMATION ON INGREDIENTS

Single Substance or Mixture Substance

Formula $C_{57}H_{82}O_{16}$

Chemical Name	Molecular weight	CAS RN	Weight-%
Ciguatoxin CTX 3C	1023.25	148471-85-6	100

Impurities and/or Additives： Not applicable

4. FIRST AID MEASURES

First aid measures

Eye contact Rinse thoroughly with plenty of water for at least 15 minutes, lifting lower and upper eyelids. Consult a physician.

Skin contact Wash skin with soap and water.

Inhalation Remove to fresh air.

Ingestion Rinse mouth. Never give anything by mouth to an unconscious person. Call a physician or poison control center immediately. Do not induce vomiting without medical advice.

Most important symptoms and effects, both acute and delayed

Symptoms No information available.

Indication of any immediate medical attention and special treatment needed

Note to physicians Treat symptomatically.

5. FIRE-FIGHTING MEASURES

Suitable Extinguishing media

Use extinguishing measures that are appropriate to local circumstances and the surrounding environment.

Unsuitable Extinguishing media Caution: Use of water spray when fighting fire may be inefficient.

Specific hazards arising from the chemical

No information available.

Explosion data

Sensitivity to Mechanical Impact none.

Sensitivity to Static Discharge none.

Protective equipment and precautions for firefighters

As in any fire, wear self-contained breathing apparatus pressure-demand, MSHA/NIOSH (approved or equivalent) and full protective gear.

6. ACCIDENTAL RELEASE MEASURES
